# Clinical Significance of HSPD1/MMP14/ITGB1/miR-6881-5P/Lnc-SPARCL1-1:2 RNA Panel in NAFLD/NASH Diagnosis: Egyptian Pilot Study

**DOI:** 10.3390/biomedicines9091248

**Published:** 2021-09-17

**Authors:** Reda Albadawy, Sara H. A. Agwa, Eman Khairy, Maha Saad, Naglaa El Touchy, Mohamed Othman, Marwa Matboli

**Affiliations:** 1 Department of Gastroentrology, Hepatology & Infectious Disease, Faculty of Medicine, Benha University, Benha 13518, Egypt; naglaa.eltoukhy@fmed.bu.edu.eg; 2 Molecular Genomics Unit, Clinical Pathology Department, Medical Ain Shams Research Institute (MASRI), School of Medicine, Ain Shams University, Cairo 11566, Egypt; 3 Medicinal Biochemistry and Molecular Biology Department, School of Medicine, Ain Shams University, Cairo 11566, Egypt; dr_emankhairy@yahoo.com; 4 Biochemistry Department, Faculty of Medicine, Modern University for Technology and Information, Cairo 11382, Egypt; maha.saad@medicine.mti.edu.eg; 5 Gastroenterology and Hepatology Section, Baylor College of Medicine, Houston, TX 77030, USA; mohamed.othman@bcm.edu

**Keywords:** NASH, NAFLD, RNA, steatohepatitis, RNA panel

## Abstract

Background: Non-alcoholic steatohepatitis ((NASH) is the progressive form of (non-alcoholic fatty liver disease) (NAFLD), which can progress to liver cirrhosis and hepatocellular carcinoma. There is no available reliable non-invasive diagnostic tool to diagnose NASH, and still the liver biopsy is the gold standard in diagnosis. In this pilot study, we aimed to evaluate the Nod-like receptor (NLR) signaling pathway related RNA panel in the diagnosis of NASH. Methods: Bioinformatics analysis was done, with retrieval of the HSPD1/MMP14/ITGB1/miR-6881-5P/Lnc-SPARCL1-1:2 RNA panel based on the relation to the NLR-signaling pathway. Hepatitis serum markers, lipid profile, NAFLD score and fibrosis score were assessed in the patients’ sera. Reverse transcriptase real time polymerase chain reaction (RT-PCR) was done to assess the relative expression of the RNA panel among patients who had NAFLD without steatosis, NAFLD with simple steatosis, NASH and healthy controls. Results: We observed up-regulation of Lnc-SPARCL1-1:2 lncRNA that led to upregulation of miR-6881-5P with a subsequent increase in levels of HSPD1, MMP14, and ITGB1 mRNAs. In addition, ROC curve analysis was done, with discriminative cutoff values that aided discrimination between NASH cases and control, and also between NAFLD, simple steatosis and NASH. Conclusion: This pilot study concluded that HSPD1/MMP14/ITGB1/miR-6881-5P/Lnc-SPARCL1-1:2 panel expression has potential in the diagnosis of NASH, and also differentiation between NAFLD, simple steatosis and NASH cases.

## 1. Introduction

Non-alcoholic steatohepatitis (NASH) is a chronic liver disease marked by the accumulation of fat in the liver (steatosis) and inflammation [1]. NASH, which is the progressive form of non-alcoholic fatty liver disease (NAFLD), is estimated to be present in one third of NAFLD cases. NASH also can progress into liver cirrhosis and hepatocellular carcinoma [2]. The progression of NAFLD to NASH is mostly attributed to a combination of genetic and environmental factors. Obesity, diabetes mellitus and hypertension are known factors to increase NASH development [3]. NASH manifests with non-specific symptoms such as fatigue and pain in the upper right abdomen, accordingly the disease may progress undetected [4].

Despite the first documentation of NASH about 40 years ago [5], its pathogenesis is still not fully understood. The proposed multiparallel hypothesis suggests that NASH is the result of multiple factors acting in parallel, including genetic predisposition, abnormal lipid metabolism, lipotoxicity, oxidative stress, mitochondrial dysfunction, altered production of cytokines and adipokines, endoplasmic reticulum stress and gut dysbiosis [6]. A prime role in NASH pathogenesis has been recently attributed to the nucleotide oligomerization domain (NOD)-like receptors (NLRs). Damage associated molecular patterns (DAMPs) or pathogen associated molecular patterns (PAMPs) that activate NLR resulting in the assembly of the inflammasome, a multiprotein complex required for caspase-1 activity and initiation of inflammatory signals. Full activation of inflammasome, mediated by pattern recognition receptors (PRRs) via NF-κB, can be induced by a broad spectrum of signals, such as uric acid, reactive oxygen species (ROS,) ATP [7] and mitochondrial DNA [8], leading to secretion of mature IL-1 [9]. These cytokines, acting on different cell types, elicit inflammatory signals in the liver as well as in the adipose tissue and intestine, triggering steatosis, insulin resistance, inflammation and cell death [10]. Inflammasomes have been recognized in NAFLD development and progression to NASH in both animal models and humans [11,12].

Abnormal miRNA expression has recently been shown to be involved in multiple diseases including metabolic diseases [13]. A growing number of deregulated miRNAs involved in synthesis of fatty acids and storage of triglycerides have been recently reported in NAFLD [14]. Examples of these miRNAs, miR-106b, miRPlus-I137, miR-1282, and miR-3663-5p were the most significantly upregulated in NAFLD, playing important roles in inflammation, insulin signaling and metabolism [15]. Additionally, microarray analysis of liver tissue from patients with NAFLD has led to the identification of 1735 lncRNAs with important roles in development and progression of NAFLD [16].

As NAFLD and NASH are asymptomatic until the advanced disease stage, the role of current treatments and life style modifications are limited [17]. Furthermore, the current gold standard in diagnosis and prognosis is the liver biopsy, which is an invasive, expensive modality with a high risk of complications and sampling errors. Thus, there is an urgent need for non-invasive, reliable, early and accurate diagnostic biomarkers. NAFLD includes both simple steatosis and NASH. Differentiation between them requires histopathological assessment. We need to establish a reliable noninvasive diagnostic model for differentiating steatosis from steatohepatitis utilizing both clinical characteristics and a panel of non-invasive RNA biomarkers through monitoring the change in the RNA differential expression among the study groups; paving the way to diagnose NASH patients [18]. In the current pilot study, we explored the NLR-signaling pathway aiming to establish a NAFLD/NASH specific mRNA-miRNA-lncRNA RNA panel to assess and validate its role in the diagnosis and stratification of NAFLD cases. Our work was reinforced by the desperate need for novel efficient diagnostic NASH biomarkers.

## 2. Materials and Methods

### 2.1. Study Population

The current study was approved by the Benha University ethical committee, faculty of medicine (approval number: MoHP0018122017, 1017). All cases were seeking medical advice in the Hepatology Clinics of Benha University Hospital from January 2021 to June 2021. There were 160 participants, with 14 NAFLD without steatosis cases, 11 NAFLD with simple steatosis cases, 55 NASH cases and 80 healthy volunteers coming for routine checkups in the hospital clinics. Written informed consent was taken from all of the study subjects.

NAFLD without steatosis, simple steatosis and NASH were diagnosed based on the clinical presentation in non-alcoholic patients with detection of steatosis by imaging after appropriate exclusion of other liver diseases, e.g., viral hepatitis and schistosomiasis, autoimmune diseases, hypertension, cardiac diseases and malignancies. There was no history of alcohol consumption in the 12 months preceding the study [19]. In addition, evaluation of steatosis score was done using ultrasonography after fasting abdominal ultrasound ((Acuson S2000, Siemens) Medical Solutions, Mountain View, California, USA) by three registered medical radiologists who graded the diagnosis into: non-steatosis (*n* = 14 cases), or mild (*n* = 11 cases), moderate (*n* = 20 cases), and severe *(n* = 25 cases) steatosis. Fibrosis staging was measured using transient elastography (Fibroscan1) classifying cases into four categories according to fibrosis score [20]. The NAFLD Fibrosis score based on several laboratory tests was used to estimate the degree of scarring in the liver [20]. Of note, the healthy control group had normal liver imaging, and normal fasting glucose levels, lipid profiles and liver enzymes.

**Figure 1:**
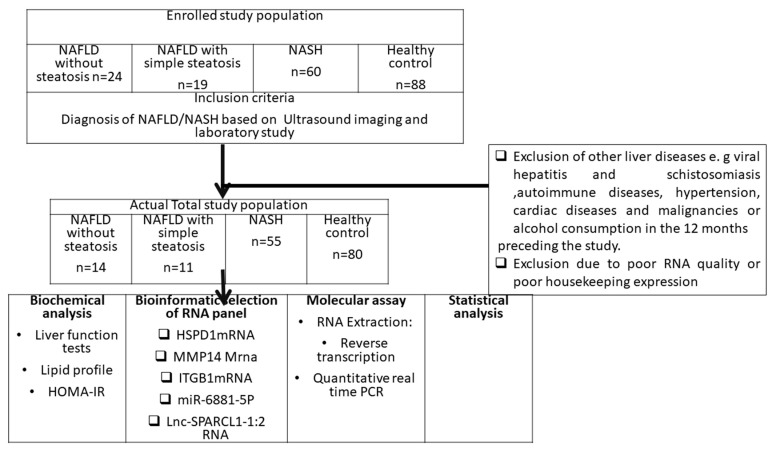
Flowchart of the study design.

All of the patients that were recruited from Hepatology Clinics of Benha University Hospital with probable NAFLD and risk factors had previous screening for bilharzial antibody, hepatitis B s antigen, hepatitis B antibodies, hepatitis C core antigen, and hepatitis C antibodies, and were excluded if they had positive results. Abdominal ultrasound was a prerequisite before enrollment in the study. The individuals from control groups were individuals seeking routine medical check-up with normal liver function tests, normal abdominal ultrasound, negative viral markers or bilharziasis and were non-alcoholic. They were age and sex matched to the patient group (Figure 1).

Blood samples were collected. Samples were centrifuged at 4000 rpm for 20 min. Then serum was preserved in aliquots in a −80 °C freezer for further processing.

AFP level was measured by using quantitative sandwich AFP ELISA Kit (My Biosource Inc., San Diego, USA). The serum ALT, AST, GGT, total bilirubin, direct bilirubin, albumin, LDL cholesterol, HDL, total cholesterol, triglycerides, fasting blood glucose, and HbA1c were measured using ALT Reagent OSR6607, aspartate aminotransferase (AST) reagent OSR6509, GGT OSR6119, total bilirubin OSR6112 reagent, direct bilirubin OSR6111, albumin OSR6102, LDL-cholesterol OSR6196, HDL-cholesterol OSR6195, total cholesterol OSR6116, triglycerides OSR60118, blood glucose OSR6121, and HbA1c OSR6192 on a multifunctional biochemistry analyzer (AU680, Beckman Coulter Inc, CA). Fasting insulin level was estimated in sera by enzyme-linked immunosorbent assay ELISA (DRG^®^ Insulin ELISA (EIA-2935), DRG International, Inc., USA). We calculated the Homeostatic Model Assessment of Insulin Resistance (HOMA-IR) by the following equation: Fasting insulin (μU/L) × fasting glucose (nmol/L)/22.5 [21].

### 2.2. Bioinformatics Set Up

At first, we reviewed the current available literature on the pathophysiology and molecular signaling pathways involved in NAFLD/NASH development [6,7,8,9,10,11,12]. Of note, there was a crucial role of the NOD-like receptors signaling pathway in the development of NAFLD/NASH. Then, based on our interest in NASH and the NLR-signaling pathway, we retrieved Heat shock protein Family D Member 1 (HSPD1, also named HSP60) mRNA, Matrix metalloproteinase 14 (MMP14) mRNA and integrin β1 (ITGB1) mRNA based on their relation to regulation of lymphocyte activation, NLR, novelty, basal expression in peripheral blood and the liver from Biosystems database (https://www.ncbi.nlm.nih.gov/Structure/biosystems/docs/biosystems_about.html, accessed at 31 July 2021), the National Center of Biotechnology Information Gene expression Omnious GEO (https://www.ncbi.nlm.nih.gov/geo/, accessed at 31 July 2021) and QuickGO databases (https://www.ebi.ac.uk/QuickGO/, accessed at 31 July 2021) (Supplementary Figure S1). The selected RNA panel was verified for their relation to NOD signaling by both the literature and KEGG map (Supplementary Figure S2). The strength of the protein to protein interaction was validated by the STRING database (Supplementary Figure S3). The MirWalk database was then used and miR-6881-5p miRNA was retrieved, interacting with the 3 mRNAs, based on novelty, high complementarity binding site numbers and novelty. The miRbase database was used to confirm the results (Supplementary Figure S4). After that, we selected the lnc-SPARCL1-1:2 lncRNA as the controller of the selected genes using the mirwalk database. At the end, sequence alignment was done between lncRNA lnc-SPARCL1-1:2(chr4:87568035-87732370, NONHSAT097317; ENST00000506480.5) and miR-6881-5p (MIMAT0027662) miRNA (Supplementary Figure S5).

### 2.3. RNA Extraction

Total RNA was extracted from the 150 μL of frozen sera samples by miRNEasy extraction kit (Qiagen, Hilden, Germany), according to the kit’s manual. RNA was eluted in a final volume of 20 μL of nuclease-free H_2_O. Concentration and purity of RNA were assessed using the Qubit TM ds DNA HS Assay Kit (Catalogue no.: Q32851) and Qubit TM RNA HS Assay Kit (Catalogue no.: Q32852) (Invitrogen by Thermo Fisher Scientific- Eugene, Oregon, USA) by Qubit 3.0 Fluorimeter (Invitrogen by life technologies, Malaysia). The samples with more than a 1.8–2 RNA:protein ratio (260:280 ratio) were considered to contain a high RNA concentration.

### 2.4. Reverse Transcription and Real-Time PCR (RT-qpcr)

We used 0.5 µg of RNA for reverse transcription by miScript II RT kit (Qiagen, Helman, Germany; Cat no. 218161) in a volume of 20 μL. RT reactions were incubated for 60 min at 37 °C, followed by 95 °C for 5 min. The relative expression of HSPD1, MMP14 and ITGB1 mRNAs were assessed by means of QuantiTect SYBR Green PCR Kit (Cat no. 204143) (Qiagen, Helman, Germany). In addition, relative expression of lncRNA lnc-SPARCL1-1:2 was assessed RT² SYBR Green ROX qPCR Master mix (Cat no: 330500; Qiagen, Helman, Germany). The PCR program for relative quantification was as follows: Initial denaturation at 95 °C for 10 min; then 45 cycles at 95 °C for 15 s; followed by annealing at 55 °C for 30 s and extension at 70 °C for 30 s. miR-6881-5p miRNA relative expression was assessed by miScript SYBR Green PCR Kit (Cat no. 218073) (Qiagen, Helman, Germany). The real-time PCR thermal cycler was programmed as follows: initial activation for 15 min at a temperature of 95 °C. Then, 40 PCR cycles were done with the setting start at 94 °C for 15 s, followed by incubation at 55 °C for 30 s and finally at 72 °C for 30 s for denaturation, annealing and extension respectively. GAPDH, SNORD72 and GAD1 were used as internal controls for the chosen mRNA, miRNA and lncRNA respectively (details in Supplementary Table S1). Samples were assessed in duplicates. Relative quantification of expression was calculated by RQ = 2^−ΔΔCt^ using the Livak method. The 7500 Fast System (applied Biosystems, Foster City, CA, USA) thermal cycler and data analyzer were used. Ct values more than 36 were considered as negative expression.

### 2.5. Statistical Analysis

To statistically analyze the data, Software Package of Statistical Analysis version 25 (SPSS25) was used. The median was used for non-parametric data (e.g., the HSPD1/MMP14/ITGB1/miR-6881-5P/Lnc-SPARCL1-1:2 RNA panel), while mean ± SD was used for symmetrically distributed raw numerical data. One-way ANOVA, cross-tabulation chi-square test for number and percentage calculation, and Spearman correlation test were used as appropriate. The receiver operating characteristic (ROC) curve was used to assess the predictive value of the RNA panel in the NASH diagnosis.

## 3. Results

### 3.1. NASH Association with Clinical and Biochemical Markers

In regards to sex, there was no significant difference between the different study groups. On the contrary, there was a significant difference in total cholesterol, HDL-cholesterol, LDL-cholesterol, total triglycerides, albumin-creatinine ratio, ALT, AST, total bilirubin, direct bilirubin, serum albumin, GGT, alfa fetoprotein, fasting blood glucose, glycated hemoglobin (HbA1C) and HOMA-IR between the NAFLD, simple steatosis, NASH and healthy control groups (*p* ˂ 0.01) (Table 1).

### 3.2. Differential Expression of the NASH Predictors in the Study Groups

**Figure 2:**
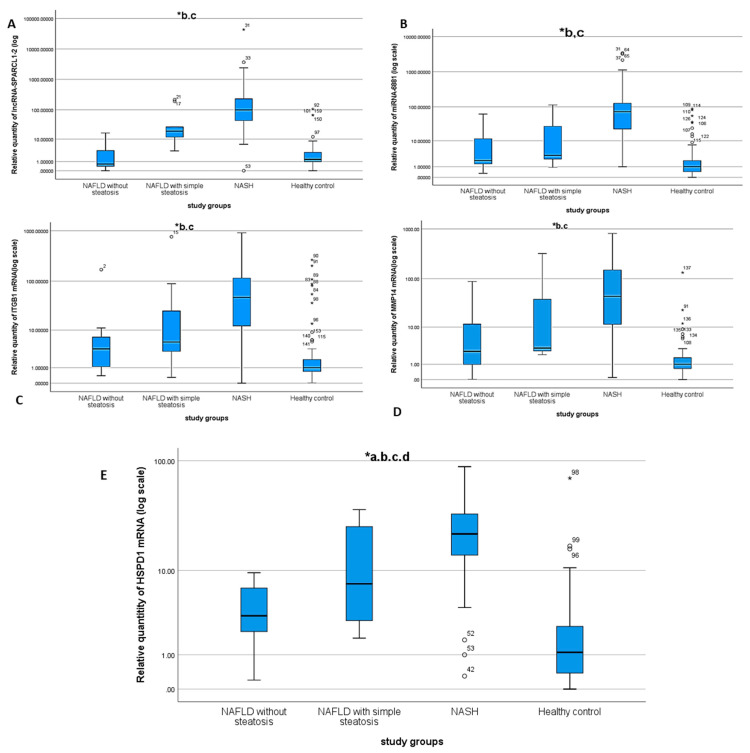
Differential expression of lnc-SPARCL1-1:2 (**A**) miR-6881-5p miRNA (**B**), ITGB1 mRNA (**C**), MMP14 mRNA (**D**) and HSPD1 mRNA (**E**) * statistically significant difference by Tukey post-hoc test in ^a^ healthy control vs. NAFLD without steatosis, ^b^ healthy control vs. NASH, ^c^ NAFLD without steatosis vs. NASH, ^d^ NASH vs. simple steatosis.

The expression of the selected RNA panel was assessed by fold change (RQ) value in the different study groups (NAFLD, simple steatosis, NASH and healthy control groups) to confirm the retrieved bioinformatics data. There was upregulation in the expression of HSPD1 mRNA, MMP14 mRNA, ITGB1 mRNA, miR-6881-5pmiRNA and lncRNA lnc-SPARCL1-1:2 in NAFLD, simple steatosis, and NASH groups compared to the healthy control group (*p* ˂ 0.01) (Figure 2A–E, Supplementary Table S2).

**Figure 3:**
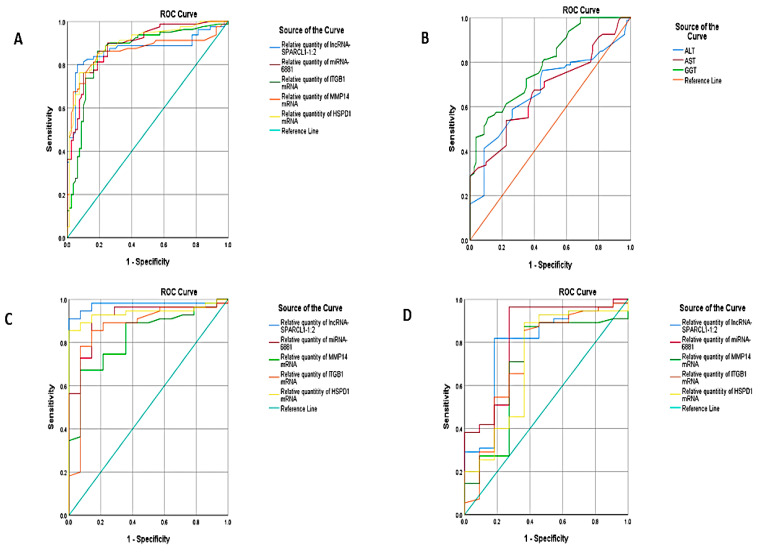
ROC curve analysis of the RNA panel between NAFLD and healthy controls (**A**), ROC curve analysis of the ALT, AST and GGT between NAFLD and healthy controls (**B**), ROC curve analysis of the RNA panel between NASH and NAFLD (**C**) and ROC curve analysis of the RNA panel between NASH and simple steatosis, (**D**) ROC curve analysis of the RNA panel between NAFLD without steatosis and NASH.

ROC curve analysis was used to compare the NASH group to the healthy control group. The best cutoff values were 2.65 for HSPD1 mRNA (AUC = 0.897), 2.05 for MMP14 mRNA (AUC = 0.862), 1.75 for ITGB1 mRNA (AUC = 0.858), 1.65 for miR-6881-5p miRNA (AUC = 0.891) and 4.45 for lncRNA lnc-SPARCL1-1:2 (AUC = 0.870). The estimated sensitivities were 88.8%, 86.3%, 90%, 90% and 83.8% respectively. The estimated specificities were 76.2%, 80%, 70%, 72.5% and 83.8% respectively. These results suggest that these best cutoff values could be used as a tool to predict and diagnose NASH cases early from healthy controls. The selected RNA panel was superior to other clinical parameters measured by non-invasive procedures like ALT, AST, and GGT (Table 2, Figure 3A,B).

ROC curve analysis was used to compare NASH with the simple steatosis group. The AUC was 0.712, 0.730, 0.695, 0.823, and 0.790, for HSPD1 mRNA, MMP14 mRNA, ITGB1 mRNA, miR-6881-5p miRNA and lncRNA lnc-SPARCL1-1:2, respectively. The estimated sensitivities were 85%, 65%, 70%, 72% and 70%, respectively. The estimated specificities were 63%, 83%, 83%, 83% and 82%, respectively. These results suggest that the selected RNA panel could be used as a tool to diagnose NASH cases from simple steatosis cases (Table 2, Figure 3C).

ROC curve analysis was used to compare the NAFLD group to the NASH group. The AUC was 0.939, 0.868, 0.825, 0.916, and 0.974, for HSPD1 mRNA, MMP14 mRNA, ITGB1 mRNA, miR-6881-5p miRNA and lncRNA lnc-SPARCL1-1:2, respectively. The estimated sensitivities were 89%, 78.2%, 74%, 70.9% and 90%, respectively. The estimated specificities were 93%, 93%, 78%, 93% and 100%, respectively. These results suggest that the selected RNA panel could be used as a tool to diagnose NASH cases from NAFLD cases (Table 2, Figure 3D).

**Figure 4:**
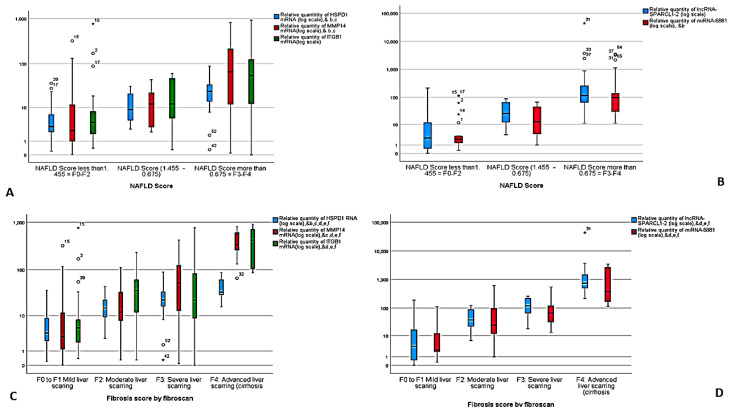
Differential expression in different NAFLD scores of HSPD1 mRNA, MMP14 mRNA, ITGB1 mRNA (**A**), miR-6881-5p miRNA and lnc-SPARCL1-1:2. * Statistically significant difference (*p* < 0.05) by Tukey post-hoc test, ^b^ NAFLD score 1 vs. 3, ^c^ NAFLD score 2 vs. 3. (**B**), and differential expression in different fibrosis scores of HSPD1 mRNA, MMP14 mRNA, ITGB1 mRNA (**C**), miR-6881-5p miRNA and lnc-SPARCL1-1:2 (**D**). * statistically significant difference (*p* < 0.05) by Tukey post-hoc test, ^b^ Fibrosis stage 2 vs. Fibrosis stage 3, ^c^ Fibrosis stage 1 vs. Fibrosis stage 3, ^d^ Fibrosis stage 1 vs. Fibrosis stage 4, ^e^ Fibrosis stage 2 vs. Fibrosis stage 4, ^f^ Fibrosis stage 3 vs. Fibrosis stage 4.

In addition, the expression pattern of the RNA panel was significantly different between NAFLD, simple steatosis, and NASH cases compared to healthy controls and also between different fibrosis scores and NAFLD scores. There was upregulation in the expression of HSPD1 mRNA by 1.3-fold in NAFLD cases compared to healthy controls, 3.5-fold in simple steatosis cases compared to NAFLD cases and 2-fold in NASH cases compared to simple steatosis cases. In addition, there was upregulation in the expression of MMP14 mRNA, ITGB1 mRNA, miR-6881-5p miRNA and lncRNA lnc-SPARCL1-1:2 by 3-fold, 1-fold, 1.6-fold and 1.8-fold, respectively, in NAFLD cases compared to healthy controls, by 5-fold, 5-fold, 3-fold and 9-fold, respectively, in simple steatosis cases compared to NAFLD cases, and by 2-fold, 1.7-fold, 11-fold and 21-fold, respectively, in NASH cases compared to simple steatosis cases (Table S1, Figure 4A–D). Thus, the chosen RNA panel may be used as a noninvasive model for distinguishing simple steatosis from NASH.

The serum level of the RNA panel was statistically correlated with the severity of liver steatosis and fibrosis. Additionally, multivariate analysis was done regarding the different predictors of NASH disease. It revealed that expression levels of HSPD1 mRNA (*p* = 0.007), MMP14 mRNA (*p* = 0.015), miR-6881-5p miRNA (*p* = 0.046) and lncRNA lnc-SPARCL1-1:2 (*p* = 0.006) were independent predictors of NASH, and NAFLD scoring steatosis grading and fibrosis scoring (*p* = 0.04) (Table 3).

### 3.3. Correlation between NASH Predictors

There was strong positive correlation between all targets selected in our RNA panel. HSPD1 mRNA, MMP14 mRNA, ITGB1 mRNA, miR-6881-5p miRNA and lncRNA lnc-SPARCL1-1:2 are all strongly positive correlated, confirming the interaction between the selected RNA network (*p* = 0.000). In addition, positive correlations were found between the RNA panel targets and ALT, AST, fasting blood glucose, HbA1C, HOMA-IR, total cholesterol and serum triglycerides (Table S3).

## 4. Discussion

The prevalence of NAFLD is increasing concomitantly with the worldwide increase in type 2 diabetes mellitus and obesity [22]. Non-alcoholic steatohepatitis (NASH), which is a subtype of NAFLD, has a potential progressive course that could result in liver fibrosis, cirrhosis, hepatocellular carcinoma and liver transplantation. It is predicated that NAFLD will soon be the main indication of liver transplantation globally. The aforementioned NASH complications have significant economic, health and patient-experience burdens on the patients and their societies [23,24]. Due to the progressive course of NASH, its early diagnosis is of high clinical importance. Unfortunately, to date there is no available non-invasive, reliable method to detect the progression of steatosis to NASH. The most accurate diagnostic test to detect NASH is still liver biopsy, which is not tolerated by most patients and clinicians due to its high cost and difficulty in repetition [25].

The liver is considered an organ of the innate immune response that filter endogenous signaling molecules and invading pathogens. Accordingly, the presence of multiple insults concomitantly activates the components of innate immunity, thus triggering chronic hepatic inflammation and steatosis, allowing the progression of NAFLD into NASH [26]. Similarly, NOD-like receptors (NLRs) had been recognized as an important aspect in NASH pathogenesis. DAMPs or PAMPs result in NLR activation with inflammasome assembly, initiating the inflammatory signaling [27]. The role of core components of the NLR signaling in NAFLD/NASH has been previously studied. Recent studies showed that lipopolysaccharide stimulation could activate the NLRP3 inflammasome in the liver, which causes increased IL-1β production [28]. Of note, inhibition of NLRP3 by thioredoxin-interacting protein is a critical player in NAFLD development in animal models [29]. Moreover, NLRP6 may play a crucial role in NASH development by inhibiting transforming growth factor-β-activated kinase 1 and NF-κB pathways [30]. NOD-like receptor C4 is closely linked with NAFLD-associated liver metastasis [31]. Another example of NLR components that is previously linked to NASH pathogenesis is arrestin domain-containing protein 3, which binds apoptosis signaling-regulating kinase 1 and mitogen-activated protein kinase [32].

In this study, we explored the NLR-signaling pathway and retrieved a NLR related molecular network, aiming to assess and validate the efficacy of the molecular network in diagnosis of NASH. Several research groups found strong upregulation of NASH gene signatures enriched for inflammatory signaling and the immune response, consistent with previous transcriptome analysis of rat and human NASH [33,34].

This pilot study design was based on stratifying patients into 3 groups; NAFLD without steatosis, NAFLD with simple steatosis and NASH. We tried to measure the change in the differential expression among the study groups. Of note, serum levels of the RNA panel were higher in participants with NAFLD. The serum level of the RNA panel strongly correlated with the severity of liver steatosis, making it a useful biomarker for NAFLD prognosis and NASH diagnosis.

Alongside the results obtained in this pilot study, many researchers have shed light on different molecular expressions in NAFLD/NASH cases. Hedjazifar et al. reported high expression of Gremlin 1 mRNA in liver biopsies of NAFLD/NASH cases. Accordingly, they proposed using Gremlin 1 as a biomarker and potential therapeutic target in complications of obesity such as NASH [35]. In addition, Nie et al. proposed that PPARγ and RARα mRNAs are down-regulated in the NAFLD rat model [36]. Similarly, Pirola et al. found that miR-122, miR-192 and miR-375 miRNAs were up-regulated in simple steatosis and even more so in NASH cases [37]. miR-451 [38] was found to be downregulated in NASH, whereas miR-34a [39] was found to be highly expressed in NASH. Moreover, NEAT1 lncRNA expression was found to be increased in the NAFLD rat model [40]. Similarly, lncRNA RP11-484N16.1 was reported to be upregulated in liver tissues of NASH patients [41].

Heat shock protein Family D Member 1 (HSPD1, also named HSP60) is a well-characterized mitochondrial chaperone that acts as a guarding system of biological activity, preventing stress-induced protein damage [42]. The main functions of HSPD1 are to preserve the integrity of cellular proteins, mainly caused by environmental changes [43], help mitochondrial replication [44], and regulate mitochondrial protein transport [45]. HSPD1 acts as a significant regulator of cytokine production and interacts with interferon regulatory factor 3 (IRF3), which is a player in the NLR and IFN-β signaling pathway [46,47,48]. Enomoto et al. reported that upregulation of HSPD1 protein inhibited the activity of mitochondrial complex IV, leading to an increase in ROS concentration in cardiac muscle [49]. Stefano et al. found no difference in the expression of HSPD1 in NASH and control groups [50]. Yuan et al. found deregulation of HSPD1 in acute hepatic injury that increased with lipid accumulation in the liver [51,52]. In our study, we found upregulation in the expression of HSPD1 mRNA with discriminative cutoff values between NASH cases and the healthy control group that could also discriminate between NAFLD without steatosis, NAFLD with simple steatosis and NASH cases.

Matrix metalloproteinases (MMPs) are a family of calcium-dependent zinc-containing endopeptidases that control ECM tissue remodeling and degradation. In normal physiological conditions, MMPs are minimally expressed, and thus homeostasis is maintained. Overexpression of MMPs leads to an imbalance between the activity of MMPs and TIMPs, which results in a variety of diseases [53,54]. MMP14 cleaves chemokines/cytokines to regulate their release and modulate IL-β activity in NLR signaling, with a distinct role in inflammation [55,56,57]. MMP14 was first described by Sato et al. as a transmembrane protein that activates pro-MMP2 to induce tumor cell invasion [58]. MMP14 is upregulated in many types of cancer, enhancing inflammation, angiogenesis, cancer cell invasion, and metastasis [59,60]. Watanabe et al. reported an increase in MMP14 mRNA level in liver fibrosis, which gradually decreased with recovery [61]. Arthur found increased levels of MMP14 in the acute phase of liver injury and with fibrosis progression [62]. In our study, we found upregulation in the expression of MMP14 mRNA with discriminative cutoff values between NASH cases and the healthy control group that could also discriminate between NAFLD without steatosis, NAFLD with simple steatosis and NASH.

ITGB1 (integrin β1), a subunit of transmembrane receptors that were formed by binding with a ITGA subunit, is associated with the phosphorylation of focal adhesion kinase (FAK) to accelerate the swift activation of downstream signaling, such as the induction of protein kinase B. The integrin subunits are specific to leukocytes that interact with complement receptor and modulate TGFβ activity [63]. There is potential cross talk between ITGB1, the NLRP3 inflammasome and TNFα in leucocytes [64]. Accordingly, a growing number of studies have revealed that ITGB1 has the potential to regulate the cell–matrix interaction, cell proliferation, spreading and metastasis [65]. Guo et al. suggested that blocking of ITGB1 could be a potential anti-inflammatory therapeutic strategy in NASH [66]. In the current study, we reported increased expression of ITGB1 mRNA in NASH. We also detected discriminative cutoff values that could discriminate between NASH cases and that healthy control group, and that could also discriminate between NAFLD without steatosis, NAFLD with simple steatosis and NASH.

MicroRNAs (miRNAs), which are a small non-coding RNAs, regulate translation and gene expression at the post-transcription level. This modulation of gene expression affects many biological functions and processes, such as glucose and lipid metabolism [67]. As miRNAs resist degradation by ribonucleases, they are very stable molecules [68]. Accordingly, circulating serum or plasma miRNAs have been proposed as attractive diagnostic tools [69]. Several research groups discussed the role of non-coding ncRNAs, e.g., miRNAs [70,71,72] and lncRNAs [73,74,75]. Huang et al. proposed that hsa-miR-6881-3p could promote osteogenic differentiation of human adipose-derived stem cells [76]. Yong et al. reported enhancement of autophagy through sponging of miR-6881 [77]. Based on the available literature, hsa-miR-6881-5p has not been correlated with NASH before. In the current study, we reported increased expression of miR-6881-5p miRNA in NASH. In addition, we detected discriminative cutoff values that could discriminate between NASH cases and healthy controls, and that could also discriminate between NAFLD without steatosis, NAFLD with simple steatosis and NASH.

Abnormal expression on lncRNAs is related to many pathological processes, including cell proliferation and differentiation, steatosis, oxidative stress and endoplasmic reticulum stress [78,79]. Multiple studies have shown that there are thousands of differentially expressed lncRNAs in NAFLD livers, unfortunately only a few of these lncRNAs have been studied [80]. Therefore, study of the role and expression of lncRNAs in NASH is worthwhile. Lnc-SPARCL1-1:2 lncRNA was not correlated with NASH before, according to the available literature. In the current study, we reported increased expression of Lnc-SPARCL1-1:2 lncRNA in NASH. In addition, we detected discriminative cutoff values that could discriminate between NASH cases and healthy controls, and that could also discriminate between NAFLD without steatosis, NAFLD with simple steatosis and NASH.

**Figure 5:**
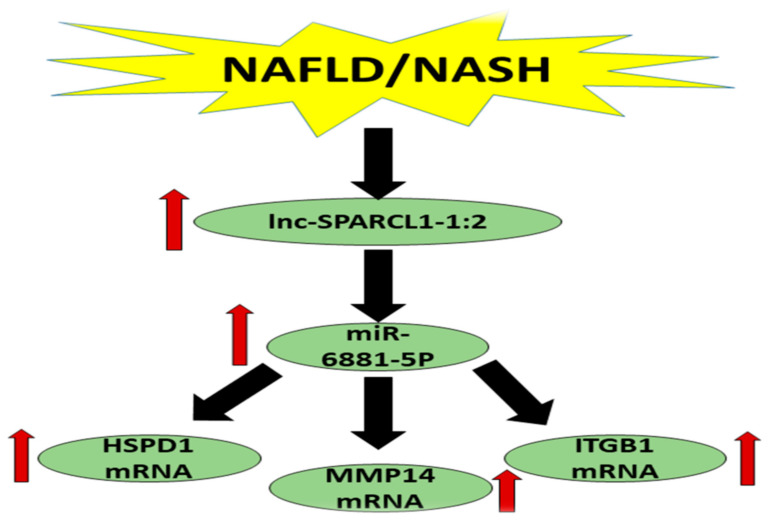
Schematic presentation of the study findings.

lncRNAs have been shown to regulate gene expression distant to the lncRNA location and act as an enhancer through DNA looping to bring the enhancer and the target site close to each other [81]. Moreover, several research works have shown that miRNAs can interact with the promoter and activate gene expression [82]. Based on the above mentioned literature and integrating it with the study statistical results, we can hypothesize that the development of NASH led to upregulation of lncRNA lnc-SPARCL1-1:2, resulting in upregulation of miR-6881-5p miRNA with a concomitant increase in the levels of HSPD1 mRNA, MMP14 mRNA and ITGB1 mRNA, confirming our bioinformatics findings and validating the role of the RNA panel in predication and early detection of NASH (Figure 5).

## 5. Conclusions

Despite the clinical importance of NASH and its growing prevalence worldwide, there is still no reliable non-invasive diagnostic tool. In this pilot preliminary study, we explored a novel RNA panel retrieved from bioinformatics databases and related to the NLR-signaling pathway, and evaluated its effectiveness in diagnosis of NASH. We concluded that HSPD1/MMP14/ITGB1/miR-6881-5P/Lnc-SPARCL1-1:2 panel expression has a potential in the diagnosis of NASH, and also in differentiation between NAFLD without steatosis, NAFLD with simple steatosis and NASH.

## Figures and Tables

**Table 1:** Demographic and laboratory data in the study groups.

Parameters	NAFLD without Steatosis *n* = 14	NAFLD with Simple Steatosis *n* = 11	NASH *n* = 55	Healthy Control *n* = 80	*p* Value
Sex	N/A				0.785
male	10 (71.4%)	7 (63.6%)	40 (72.7%)	52 (65%)	
female	4 (28.6%)	4 (36.4%)	15 (27.3%)	18 (35%)	
History of diabetes mellitus	N/A	N/A	N/A	N/A	0.00 **
positive	13 (92.9%)	9 (81.8%)	46 (83.6%)	27 (33.8%)	
negative	1 (7.1%)	2 (18.2%)	9 (16.4%)	53 (66.3%)	
Body mass index (kg/m^2^) BMI	35.7 ± 4.2	35.4 ± 4.2	35.11 ± 6.1	25.99 ± 3.2	^a^ 0.00 ^b^ 0.00 ^c^ 0.677
Total cholesterol (mg/dL)	285.14 ± 35.4	291.36 ± 43.2	300.91 ± 68.7	176.63 ± 84.9	^a^ 0.00 ^b^ 0.00 ^c^ 0.479
LDLc (mg/dL)	200.14 ± 32.3	202.73 ± 35.2	212.31 ± 60.5	126.15 ± 64.5	^a^ 0.00 ^b^ 0.00 ^c^ 0.495
HDLc (mg/dL)	34.64 ± 10	28.82 ± 7.3	28.80 ± 8.1	53.49 ± 20.9	^a^ 0.00 ^b^ 0.00 ^c^ 0.226
Total triglycerides (mg/dL)	259.29 ± 76	268.18 ± 61.7	288.45 ± 74.8	163.91 ± 92.6	^a^ 0.00 ** ^b^ 0.00 ** ^c^ 0.247
albumin creatinie ratio	23.43 ± 4.2	25.27 ± 4.38	24.22 ± 4.9	19.04 ± 6.8	^a^ 0.011 * ^b^ 0.00 ** ^c^ 0.655
AST (IU/L)	58.85 ± 20.9	69.98 ± 39.3	74.43 ± 43.9	48.13 ± 18.9	^a^ 0.241 ^b^ 0.00 ** ^c^ 0.1
ALT (IU/L)	37.21 ± 20	47.300 ± 24.28	57.78 ± 41.28	33.96 ± 16.4	^a^ 0.692 ^b^ 0.00 ** ^c^ 0.016 *
Total bilirubin (mg/dL)	2.01 ± 0.6	2.74 ± 0.4	2.9 ± 1	1.57 ± 1.3	^a^ 0.187 ^b^ 0.00 ** ^c^ 0.005 **
Direct bilirubin (mg/dL)	1.21 ± 0.5	1.66 ± 0.2	1.7 ± 0.7	0.90 ± 0.4	^a^ 0.07 ^b^ 0.00 ** ^c^ 0.005 **
Albumin (g/dL)	3.14 ± 0.2	2.48 ± 0.4	2.3 ± 0.4	3.23 ± 0.3	^a^ 0.443 ^b^ 0.00 ** ^c^ 0.00 **
Gamma glutammyl transferase (IU/L)	38.73 ± 20.2	46 ± 26.4	73.9 ± 44.46	26.7 ± 20.76	^a^ 0.187 ^b^ 0.00 ** ^c^ 0.00 **
alpha fetoprotein	81.17 ± 85.4	160.13 ± 124.67	341.85 ± 534	17.26 ± 30.8	^a^ 0.448 ^b^ 0.00 ** ^c^ 0.007 **
Fasting blood glucose (mg/dL)	213.71 ± 87.5	183.18 ± 80.3	186.27 ± 83.7	143.02 ± 87	^a^ 0.005 ** ^b^ 0.004 ** ^c^ 0.286
Glycated hemoglobin HbA1c (%)	6.32 ± 2.4	6.72 ± 1.7	7.7 ± 1.7	6.2 ± 2.7	^a^ 0.496 ^b^ 0.00 ** ^c^ 0.039 *
HOMA IR	6.5 ± 7.8	16.5 ± 8.1	18.15 ± 6.7	4.4 ± 6.1	^a^ 0.272 ^b^ 0.00 ** ^c^ 0.00 **
Scoring and Grading					
NAFLD Score	N/A	N/A	N/A	---	---
NAFLD Score < −1.455 = F0F2	13 (92.9%)	7 (63.6%)	1 (1.8%)		
NAFLD Score −1.455–0.675	1 (7.1%)	3 (27.3%)	6 (10.9%)		
NAFLD Score > 0.675 = F3F4	0 (0.0%)	1 (2%)	48 (98%)		
Fbrosis score	N/A	N/A	N/A	---	---
F0 to F1 Mild liver scaring	14 (100%)	8 (72.7%)	3 (5.5%)		
F2: Moderate liver scarring	0 (0%)	2 (18.2%)	18 (32.7%)		
F3: Severe liver scarring	0 (0%)	0 (0%)	24 (43.6%)		
F4: Advanced liver scarring (cirrhosis	0 (0%)	0 (0%)	10 (18.2%)		
steatosis grading	N/A	N/A	N/A	---	---
S1 mild steatosis	0 (0.0%)	10 (90.9%)	1 (1.8%)		
S2 moderate stetosis	0 (0.0%)	1 (9.1%)	19 (34.5%)		
S3 severe steatosis	0 (0.0%)	0 (0.0%)	25 (63.6%)		
S4 non steatosis	14 (100%)	0 (0.0%)	0 (0.0%)		

One-way ANOVA was performed to determine the differences among the study groups. Abbreviation: BMI = body mass index, FBS = fasting blood sugar, HDL-C = high density lipoprotein cholesterol, LDL-C = low density lipoprotein cholesterol, GGT = gamma glutamyl transferase, AST = aspartate transaminase, ALT = alanine transaminase, ^a^ healthy control vs. NAFLD, ^b^ healthy control vs. NASH, ^c^ NAFLD vs. NASH ** *p* < 0.01; * *p* < 0.05 (One-way ANOVA with Tukey post-hoc test).

**Table 2:** Performance characteristics of the investigated RNA panel.

Test Result Variable(s)	Area under Curve	Std. Error	Asymptotic Sig	Asymptotic 95% Confidence Interval				
				Lower Bound	Upper Bound	Cutoff	Sensitivity	Specificity
NAFLD versus healthy control								
Relative quantity of lncRNA-SPARCL1-1:2	0.870	0.032	0.000	0.807	0.932	4.45	83.8%	83.8
Relative quantity of miRNA-6881	0.891	0.025	0.000	0.842	0.940	1.65	90%	72.5%
Relative quantity of ITGB1 mRNA	0.858	0.032	0.000	0.796	0.920	1.75	90%	70%
Relative quantity of MMP14 mRNA	0.862	0.033	0.000	0.798	0.925	2.05	86.3	80
Relative quantity of HSPD1 mRNA	0.897	0.025	0.000	0.847	0.947	2.65	88.8	76.2
ALT	0.674	0.043	0.000	0.589	0.759	42	55%	74%
AST	0.674	0.043	0.000	0.590	0.757	57	53.8%	77.5%
GGT	0.790	0.035	0.000	0.722	0.858	42	61.3%	82%
NASH versus NAFLD								
Relative quantity of lncRNA-SPARCL1-1:2	0.974	0.019	0.000	0.936	1.000	17.2	90%	100
Relative quantity of miRNA-6881	0.916	0.040	0.000	0.838	0.994	24.8	70.9%	93.2
Relative quantity of ITGB1 mRNA	0.825	0.056	0.000	0.715	0.936	12.5	74%	78%
Relative quantity of MMP14 mRNA	0.868	0.059	0.000	0.752	0.984	11.3	78.2%	78%
Relative quantity of HSPD1 mRNA	0.939	0.029	0.000	0.883	0.995	5.3	89%	93%
NASH versus Simple steatosis								
Relative quantity of lncRNA-SPARCL1-1:2	0.790	0.079	0.003	0.636	0.945	54.5	70%	81%
Relative quantity of miRNA-6881	0.823	0.077	0.001	0.672	0.974	24.6	72%	83%
Relative quantity of ITGB1 mRNA	0.695	0.095	0.042	0.508	0.882	12.1	70%	83%
Relative quantity of MMP14 mRNA	0.730	0.094	0.017	0.545	0.914	20.3	65%	83%
Relative quantity of HSPD1 mRNA	0.712	.096	0.027	0.525	0.900	9.4	85.5%	63%

**Table 3:** Showing multivariate regression analysis.

Predictors	Sig.	Exp(B)	95% C.I. for EXP(B)	
			Lower	Upper
sex	0.683	1.254	0.423	3.713
NAFLD scoring	0.000	54.828	9.483	317.015
grading of steatosis	0.051	0.943	0.891	0.922
fibrosis scoring	0.04	0.955	0.877	0.891
lncRNA-SPARCL1-1:2	0.006	0.958	0.929	0.988
miRNA-6881	0.046	0.964	0.930	0.999
ITGB1 mRNA	0.087	1.014	0.998	1.029
MMP14 mRNA	0.015	0.965	0.937	0.993
HSPD1 mRNA	0.007	0.924	0.873	0.979

## Data Availability

The data reported in this study are available on request from the corresponding authors.

## Supplementary Materials

The following are available online at https://www.mdpi.com/article/10.3390/biomedicines9091248/s1, Figure S1: Validation of the relation between HSPD1, MMP14 and ITGB1 genes to NAFLD/NASH pathogenesis, B cell proliferation/Cytokine response by public microarray databases, Figure S2: Validation of the relation between HSPD1, MMP14 and ITGB1 genes to B cell proliferation/Cytokine response and NLR signaling by linking to KEGG map database, Figure S3: Validation of the interaction between HSPD1, MMP14 and ITGB1 in STRING database, Figure S4: Validation of the interaction between the selected mRNAs and the retrieved hsa-miR-6881-5p from mirWalk database, target scan database and EBI database, Figure S5: Validation of the interaction between the retrieved hsa-miR-6881-5p and lnc-SPARCL1-1:2 lncRNA, Table S1: List of primer assays, Table S2: Differential expression of blood based RNA panel among the study groups, Table S3: correlation between the expressions of different laboratory parameters among the investigated groups.
