# Supplementary material for: Clinical Significance of HSPD1/MMP14/ITGB1/miR-6881-5P/Lnc-SPARCL1-1:2 RNA Panel in NAFLD/NASH Diagnosis: Egyptian Pilot Study
Source: Biomedicines. 2021 Sep 17;9(9):1248. doi: 10.3390/biomedicines9091248 (PMC8472260; doi:10.3390/biomedicines9091248)

Article

Clinical Significance of HSPD1/MMP14/ITGB1/miR-6881-5P/Lnc-SPARCL1-1:2 RNA panel in NAFLD/NASH Diagnosis: Egyptian Pilot Study

Reda Albadawy [1]*, Sara HA. Agwa* [2], Eman Khairy [3], Maha Saad[4], Naglaa El touchy[1], Mohamed Othman[5] and Marwa Matboli[3]*

[1] Department of Gastroentrology, Hepatology &Infectious disease, Faculty of Medicine, Benha University Benha, Egypt, reda.albadawy@fmed.bu.edu.eg, naglaa.eltoukhy@fmed.bu.edu.eg

[2] Clinical Pathology Department, Molecular Genomics Unit of Medical Ain Shams Research Institute , School of Medicine, Ain Shams University; Cairo, Egypt, sara.h.agwa@med.asu.edu.eg

[3] Medicinal Biochemistry and Molecular biology Department, Ain Shams University School of Medicine, Cairo, Egypt, dr_emankhairy@yahoo.com , Marwasayed472@yahoo.com , DrMarwa__Matboly@med.asu.edu.eg.

[4] Biochemistry Department, Faculty of Medicine, Modern University for Technology and Information; Cairo, Egypt, mahasaad9292@gmail.com, maha.saad@medicine.mti.edu.eg

[5] Gastroenterology and Hepatology Section Baylor College of Medicine, Houston, Texas, USA, mohamed.othman@bcm.edu

* Correspondence: authors: Marwa Matboli[2], Medicinal Biochemistry Department, Ain Shams University School of Medicine. Marwasayed472@yahoo.com, DrMarwa__Matboly@med.asu.edu.eg, Sara HA. Agwa, Clinical pathology, Medical Ain Shams Research institute (MASRI) Cairo, Egypt.sarakariem@gmail.com.

Supplementary tables

Table S1. : List of primer assays.

Primer Assay	GeneGlobe ID
HSPD1 (XR_037196)	QT01670291
MMP14 (NM_004995)	QT00001533
ITGB1 (NM_004763)	QT00024654
GAPDH (NM_001256799)	QT00079247
Hs_miR-6881-5p_1	MS00048069
Hs_SNORD72_11	MS00033719
SPARCL1-1:2 (ENST00000506480)	SBH0300480
HS_GAD1_1390172	SBH0146388

Table S2. Differential expression of blood based RNA panel among the study groups.

study groups		lncRNA-SPARCL1-1:2	miRNA-6881	ITGB1 mRNA	MMP14 mRNA	HSPD1 mRNA
NAFLD	Mean	5.74	9.05	15.83	10.99	4.02
	Std. Deviation	16.78	16.60	44.48	22.91	2.85
	Median	1.35	2.0	3.7	2.65	3.4

Simple steatosis	Mean	50	29.0	84.45	54.70	13.91
	Std. Deviation	75.64	45.4	225.8	100.60	13.62
	Median	19.03	3.2	5.4	3.2	7.40
NASH	Mean	1053.34	327.07	143.61	132.26	27.44
	Std. Deviation	5815.49	789.31	236.69	195.9	20.20
	Median	98.0	72.2	47.3	44.0	22.0
Healthy control	Mean	3.14	5.61	11.9	3.5	3.1
	Std. Deviation	4.82	15.39	40.81	15.	8.19
	Median	.65	1.	1.0	1.0	1.1

Table S3: correlation between the expressions of different laboratory parameters among the investigated groups

			lncRNA-SPARCL 1-1:2	miRNA-6881	ITGB1 mRNA	MMP14 mRNA	HSPD1 mRNA
Spearman's rho	lncRNA-SPARCL1-1:2	Correlation Coefficient	1.000	.698	.597	.686	.664
		Sig. (2-tailed)	.	.000	.000	.000	.000
	miRNA-6881	Correlation Coefficient	.698	1.000	.599	.656	.682
		Sig. (2-tailed)	.000	.	.000	.000	.000
	ITGB1 mRNA	Correlation Coefficient	.597	.599	1.000	.646	.687
		Sig. (2-tailed)	.000	.000	.	.000	.000
	MMP14 mRNA	Correlation Coefficient	.686	.656	.646	1.000	.652
		Sig. (2-tailed)	.000	.000	.000	.	.000
	HSPD1 mRNA	Correlation Coefficient	.664	.682	.687	.652	1.000
		Sig. (2-tailed)	.000	.000	.000	.000	.
	ALT	Correlation Coefficient	.210	.233	.225	.157	.130
		Sig. (2-tailed)	.008	.003	.004	.047	.100
	AST	Correlation Coefficient	.181	.180	.245	.113	.192
		Sig. (2-tailed)	.022	.023	.002	.156	.015
	Fasting blood glucose	Correlation Coefficient	.215	.265	.265	.200	.296
		Sig. (2-tailed)	.006	.001	.001	.011	.000
		N	160	160	160	160	160
	HbA1c	Correlation Coefficient	.274	.285	.300	.236	.274
		Sig. (2-tailed)	.000	.000	.000	.003	.000
	HOMA_IR	Correlation Coefficient	.553	.563	.469	.479	.636
		Sig. (2-tailed)	.000	.000	.000	.000	.000
	Total Cholesterol	Correlation Coefficient	.461	.509	.434	.405	.506
		Sig. (2-tailed)	.000	.000	.000	.000	.000
	TGs	Correlation Coefficient	.457	.465	.410	.347	.470
		Sig. (2-tailed)	.000	.000	.000	.000	.000

Spearman correlation -test was performed to determine the differences among the the study groups. Abbreviation: , FBS = fasting blood sugar,, HDL-C = high density lipoprotein cholesterol, LDL-C = low density lipoprotein cholesterol, GGT= Gamma glutamyl transferase,, AST = aspartate transaminase, ALT = alanine transaminase

Supplementary figures:

Figures S1: Validation of the relation between HSPD1, MMP14 & ITGB1 genes to NAFLD/NASH pathogenesis, B cell proliferation/Cytokine response by public microarray databases

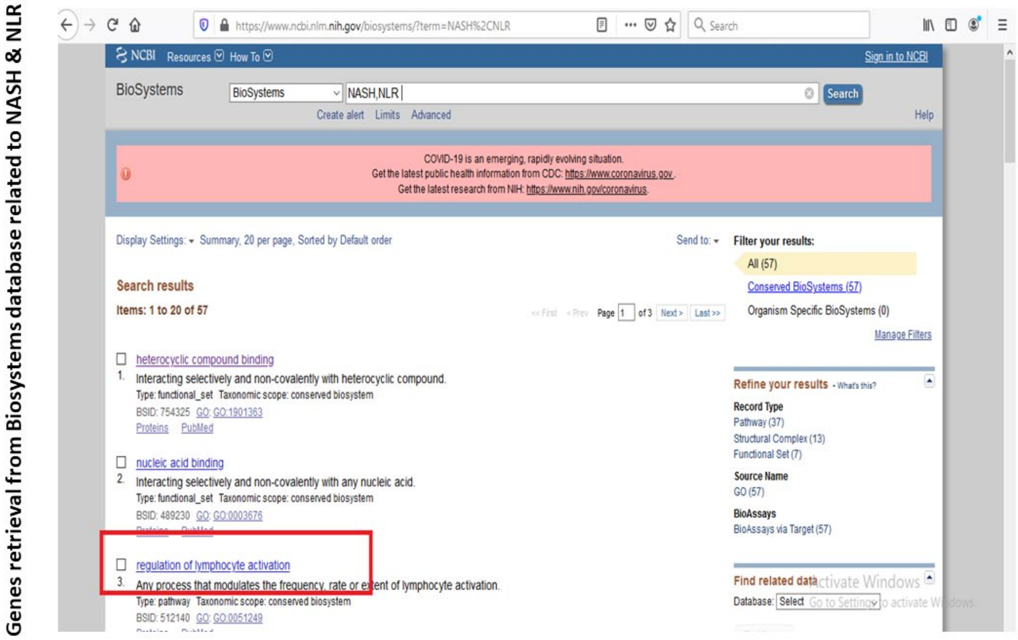

HSPD1 gene expression in NAFLD/NASH

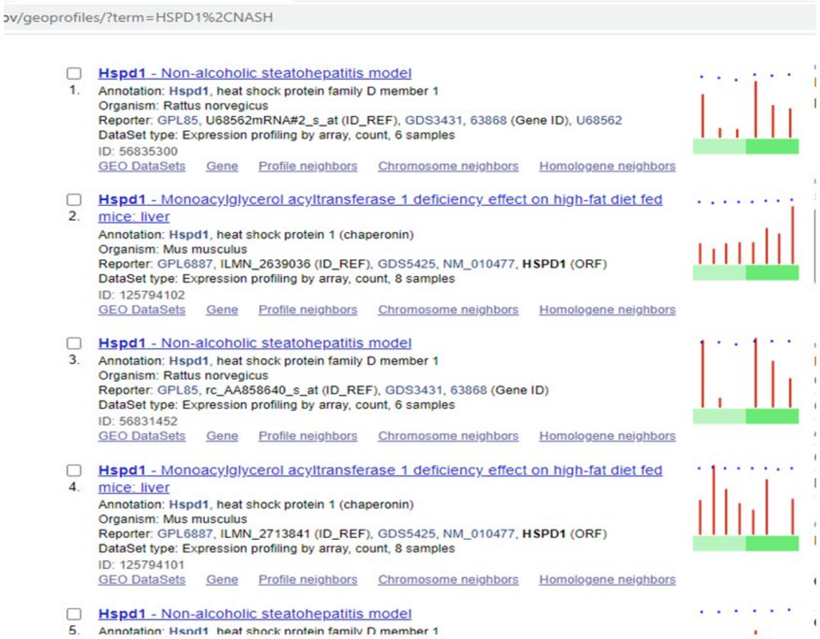

HSPD1 gene ontology

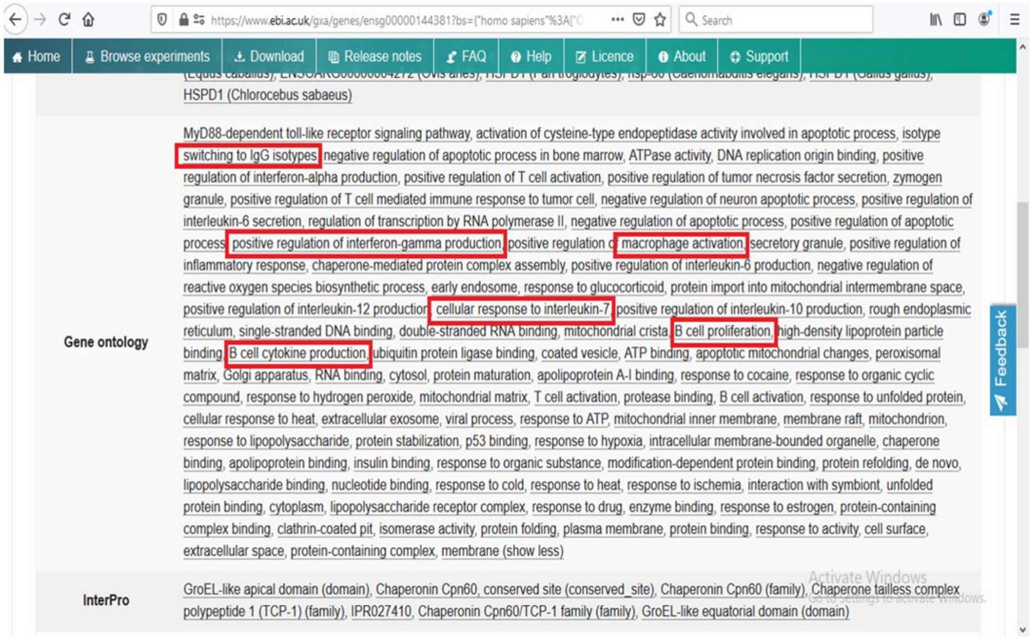

HSPD1 gene expression in different tissues especially liver

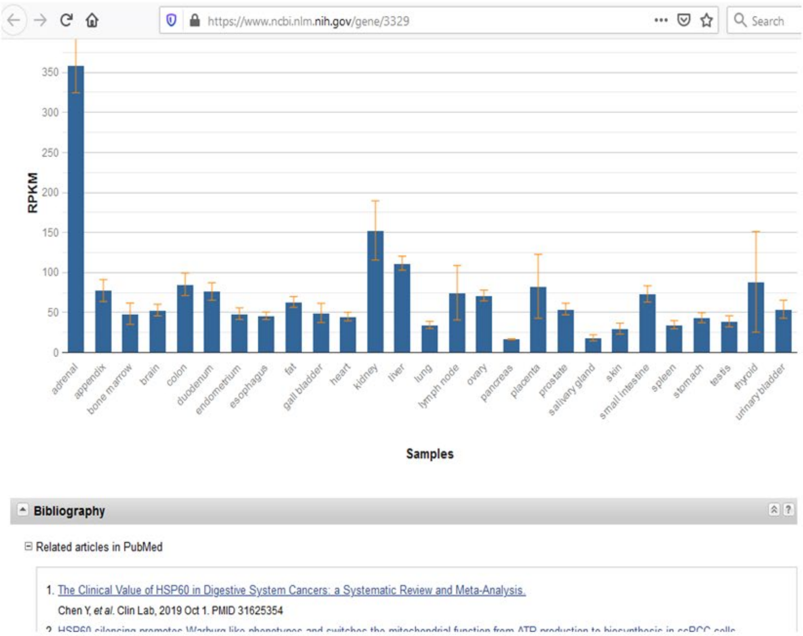

MMP14 gene expression in NAFLD/NASH

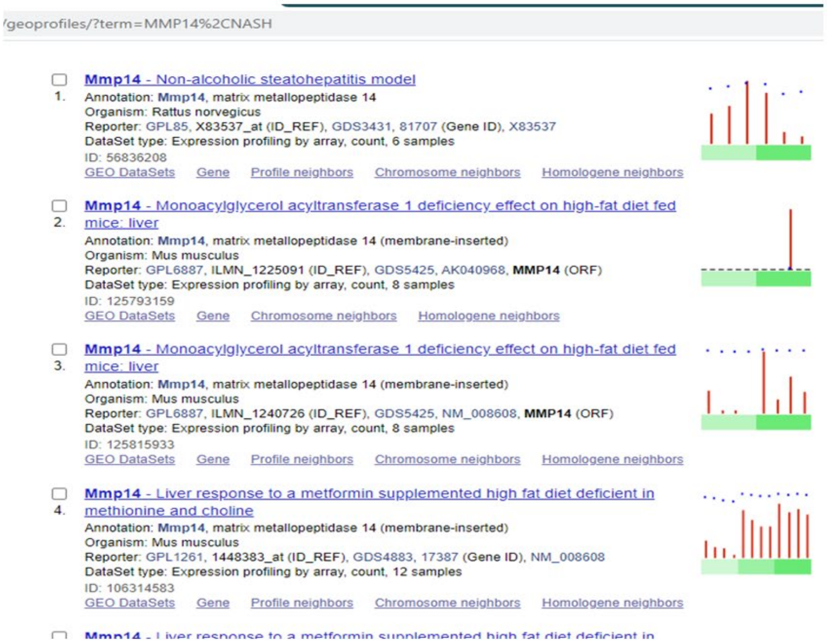

MMP14 gene ontology

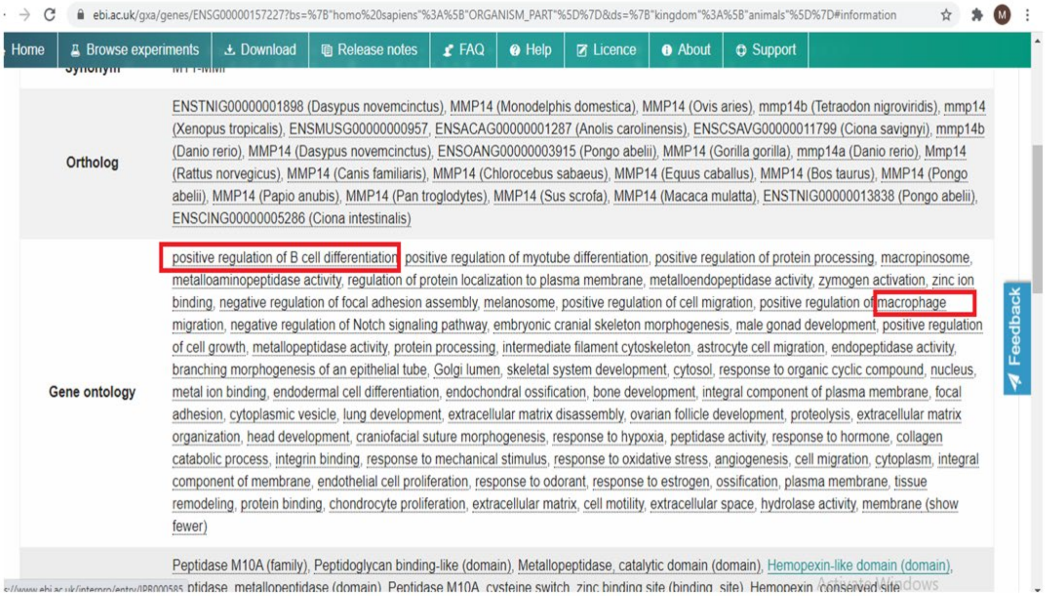

ebi.ac.uk/gxa/genes/ENSG00000157227?bs=%7B"homo%20sapiens"%3A%5B"ORGANISM_PART"%5D%7D&ds=%7B"kingdom"%3A%5B"animals"%5D%7D#information

Home | Browse experiments | Download | Release notes | FAQ | Help | Licence | About | Support

Ortholog	ENSTNIG00000001898 (Dasypus novemcinctus), MMP14 (Monodelphis domestica), MMP14 (Ovis aries), mmp14b (Tetraodon nigroviridis), mmp14 (Xenopus tropicalis), ENSMUSG00000000957, ENSACAG00000001287 (Anolis carolinensis), ENSCSAVG00000011799 (Ciona savignyi), mmp14b (Danio rerio), MMP14 (Dasypus novemcinctus), ENSOANG00000003915 (Pongo abelii), MMP14 (Gorilla gorilla), mmp14a (Danio rerio), Mmp14 (Rattus norvegicus), MMP14 (Canis familiaris), MMP14 (Chlorocebus sabaeus), MMP14 (Equus caballus), MMP14 (Bos taurus), MMP14 (Pongo abelii), MMP14 (Papio anubis), MMP14 (Pan troglodytes), MMP14 (Sus scrofa), MMP14 (Macaca mulatta), ENSTNIG00000013838 (Pongo abelii), ENSCING00000005286 (Ciona intestinalis)
Gene ontology	positive regulation of B cell differentiation, positive regulation of myotube differentiation, positive regulation of protein processing, macropinosome, metalloaminopeptidase activity, regulation of protein localization to plasma membrane, metalloendopeptidase activity, zymogen activation, zinc ion binding, negative regulation of focal adhesion assembly, melanosome, positive regulation of cell migration, positive regulation of macrophage migration, negative regulation of Notch signaling pathway, embryonic cranial skeleton morphogenesis, male gonad development, positive regulation of cell growth, metallopeptidase activity, protein processing, intermediate filament cytoskeleton, astrocyte cell migration, endopeptidase activity, branching morphogenesis of an epithelial tube, Golgi lumen, skeletal system development, cytosol, response to organic cyclic compound, nucleus, metal ion binding, endodermal cell differentiation, endochondral ossification, bone development, integral component of plasma membrane, focal adhesion, cytoplasmic vesicle, lung development, extracellular matrix disassembly, ovarian follicle development, proteolysis, extracellular matrix organization, head development, craniofacial suture morphogenesis, response to hypoxia, peptidase activity, response to hormone, collagen catabolic process, integrin binding, response to mechanical stimulus, response to oxidative stress, angiogenesis, cell migration, cytoplasm, integral component of membrane, endothelial cell proliferation, response to odorant, response to estrogen, ossification, plasma membrane, tissue remodeling, protein binding, chondrocyte proliferation, extracellular matrix, cell motility, extracellular space, hydrolase activity, membrane (show fewer)
	Peptidase M10A (family), Peptidoglycan binding-like (domain), Metallopeptidase, catalytic domain (domain), Hemopexin-like domain (domain), [illegible]ptidase, metallopeptidase (domain), Peptidase M10A, cysteine switch, zinc binding site (binding site), Hemopexin, conserved site

Feedback

MMP14 gene expression in different tissues especially liver

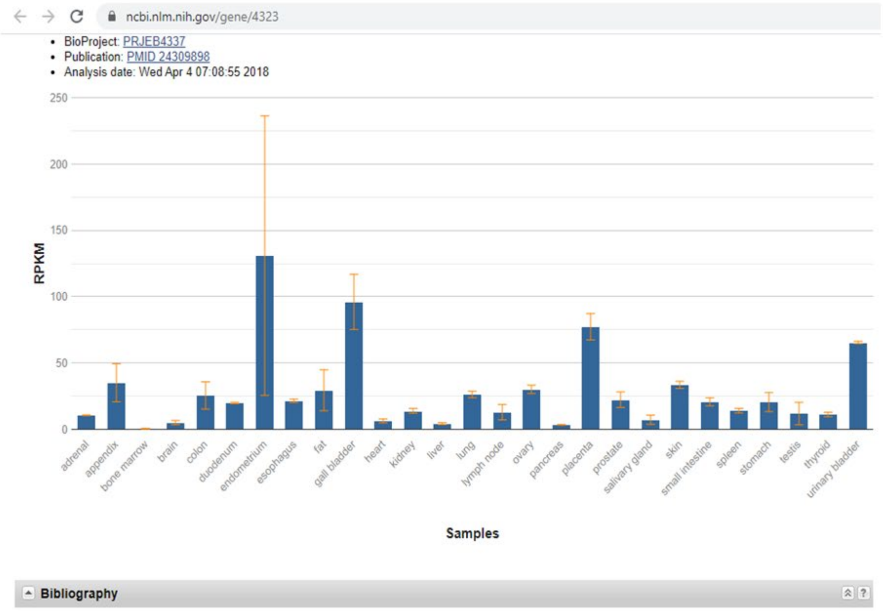

ITGB1 gene expression in NAFLD/NASH

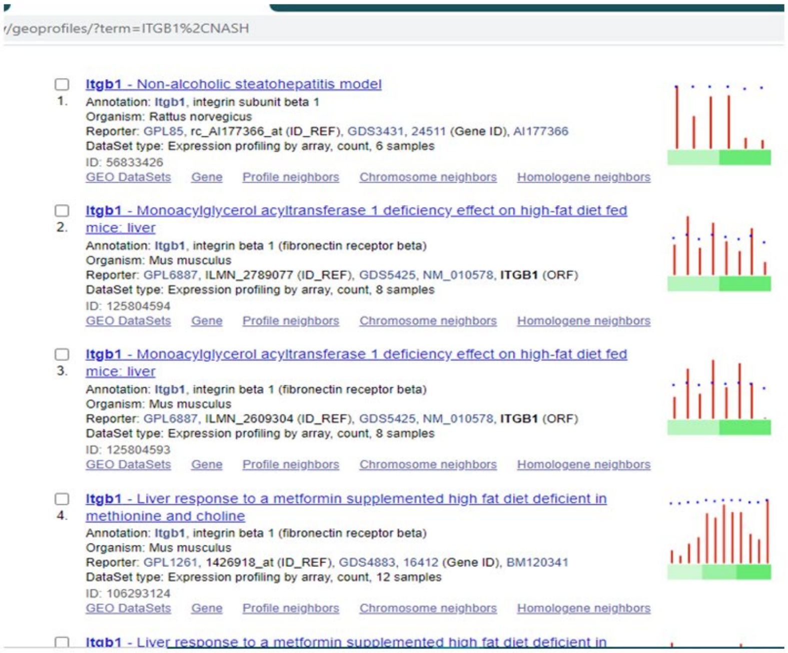

ITGB1 gene ontology

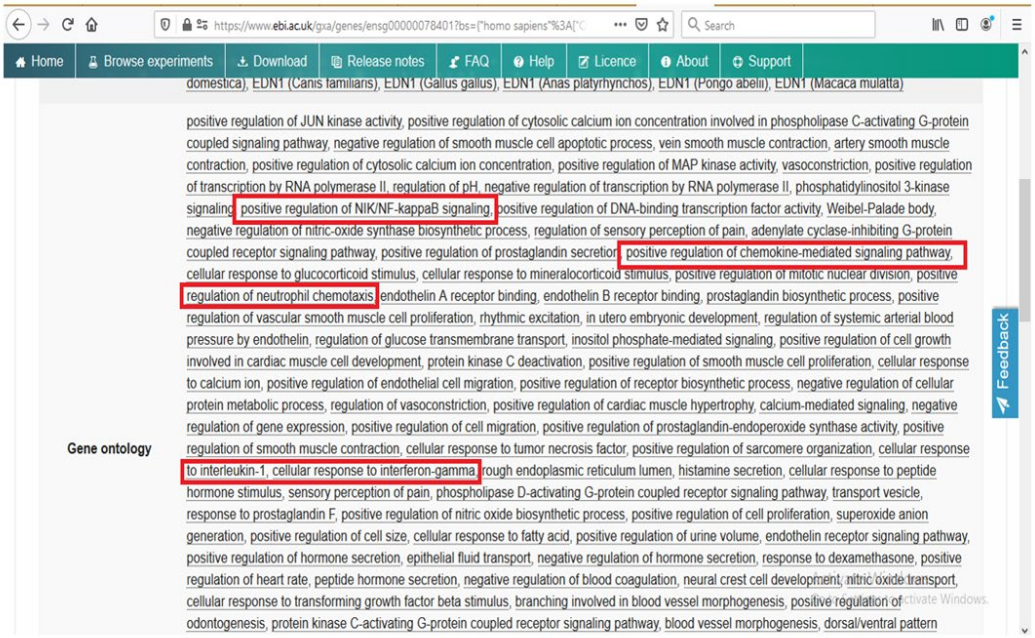

ITGB1 gene expression in different tissues especially liver

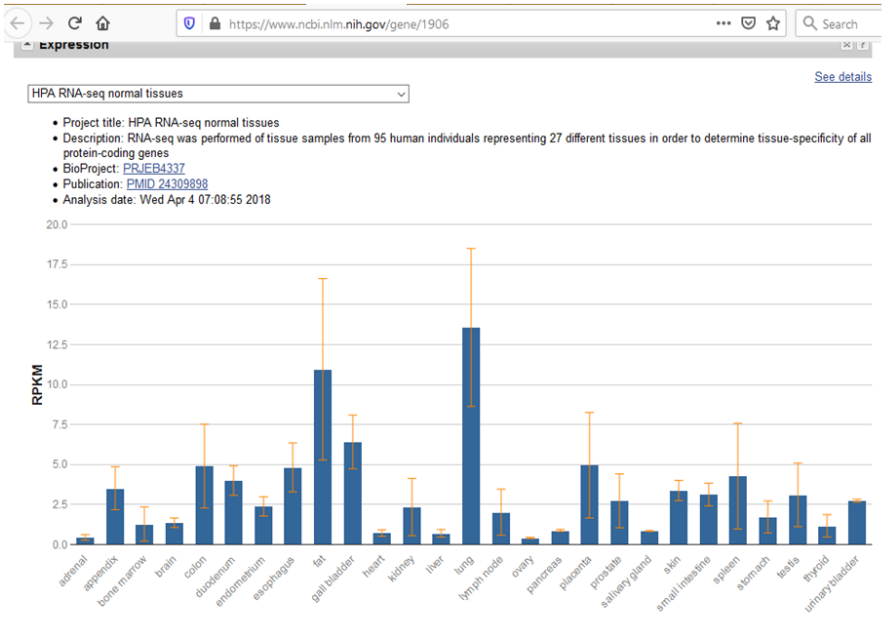

Figure S2:Validation of the relation between HSPD1, MMP14 & ITGB1 genes to B cell proliferation/Cytokine response and NLR signalling by linking to KEGG map database

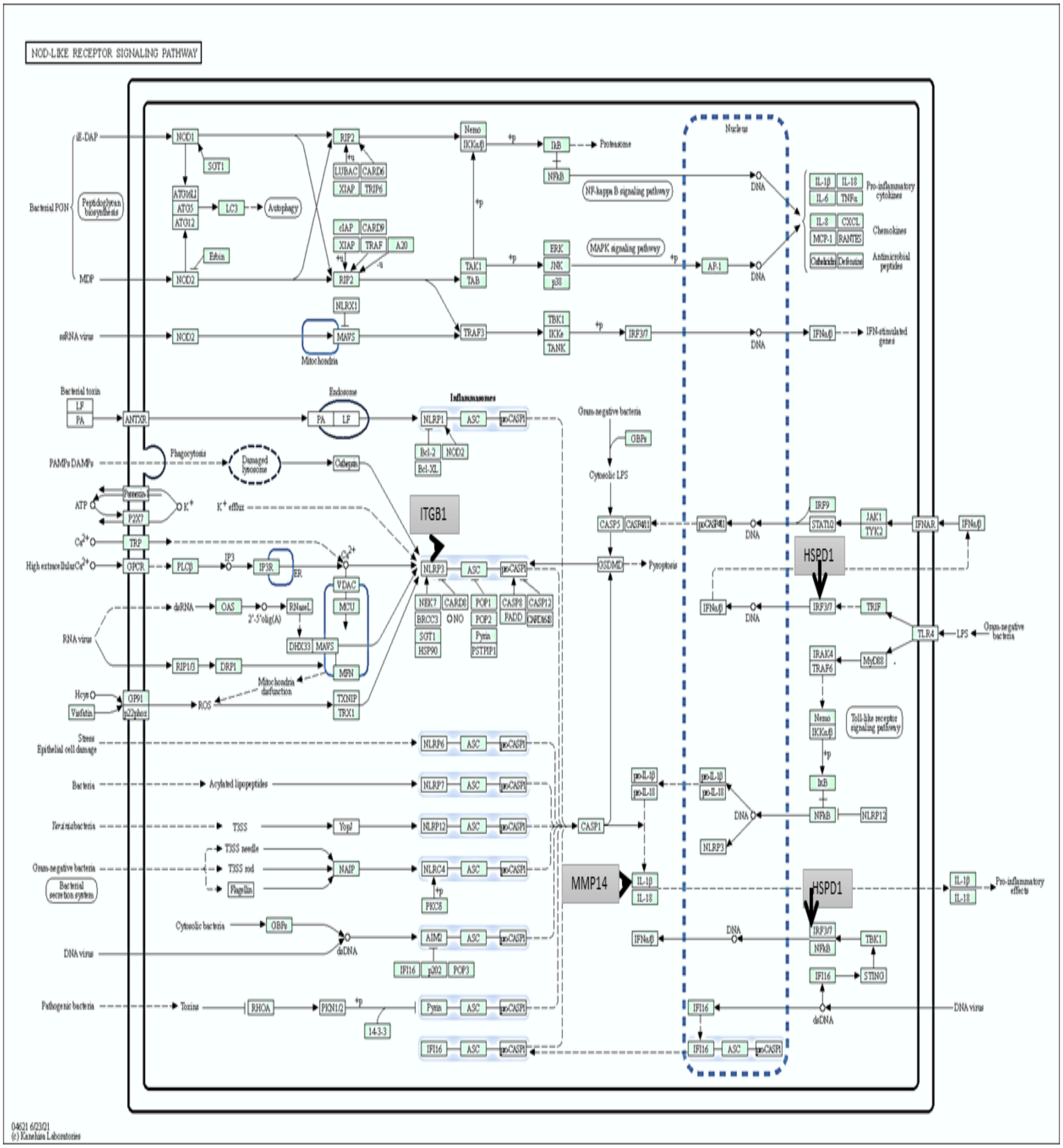

Figure S3: Validation of the interaction between HSPD1, MMP14 & ITGB1 in STRING database

Validation of the association between HSPD1, MMP14 & ITGB1 by String database

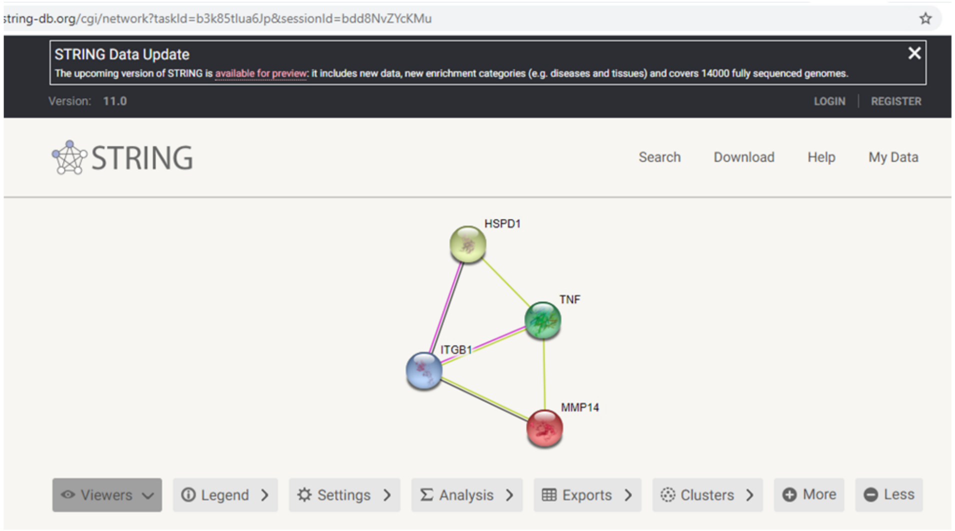

Figure S4: Validation of the interaction between the selected mRNAs and the retrieved hsa-miR-6881-5p from mirWalk database, target scan database and EBI database

hsa-miR-6881-5p target the selected mRNAs

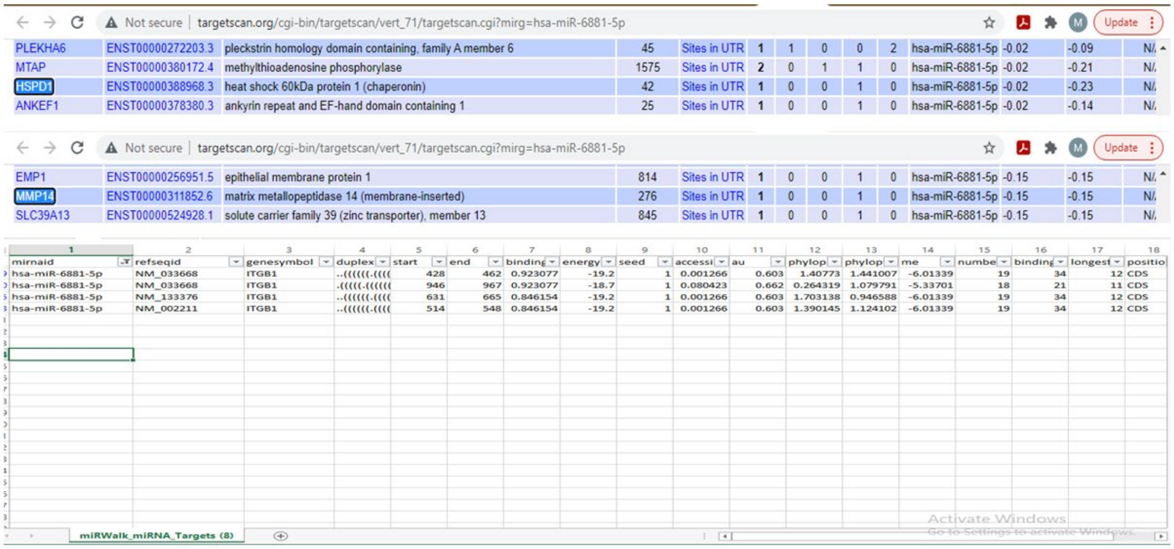

targetscan.org/cgi-bin/targetscan/vert_71/targetscan.cgi?mirg=hsa-miR-6881-5p

PLEKHA6	ENST00000272203.3	pleckstrin homology domain containing, family A member 6	45	Sites in UTR	1	1	0	0	2	hsa-miR-6881-5p	-0.02	-0.09	N/.
MTAP	ENST00000380172.4	methylthioadenosine phosphorylase	1575	Sites in UTR	2	0	1	1	0	hsa-miR-6881-5p	-0.02	-0.21	N/.
HSPD1	ENST00000388968.3	heat shock 60kDa protein 1 (chaperonin)	42	Sites in UTR	1	0	0	1	0	hsa-miR-6881-5p	-0.02	-0.23	N/.
ANKEF1	ENST00000378380.3	ankyrin repeat and EF-hand domain containing 1	25	Sites in UTR	1	0	0	1	0	hsa-miR-6881-5p	-0.02	-0.14	N/.

targetscan.org/cgi-bin/targetscan/vert_71/targetscan.cgi?mirg=hsa-miR-6881-5p

EMP1	ENST00000256951.5	epithelial membrane protein 1	814	Sites in UTR	1	0	0	1	0	hsa-miR-6881-5p	-0.15	-0.15	N/.
MMP14	ENST00000311852.6	matrix metallopeptidase 14 (membrane-inserted)	276	Sites in UTR	1	0	0	1	0	hsa-miR-6881-5p	-0.15	-0.15	N/.
SLC39A13	ENST00000524928.1	solute carrier family 39 (zinc transporter), member 13	845	Sites in UTR	1	0	0	1	0	hsa-miR-6881-5p	-0.15	-0.15	N/.

mirnaid	refseqid	genesymbol	duplex	start	end	binding	energy	seed	accessi	au	phylop	phylop	me	numbe	binding	longest	positio
hsa-miR-6881-5p	NM_033668	ITGB1	..((((((.((((	428	462	0.923077	-19.2	1	0.001266	0.603	1.40773	1.441007	-6.01339	19	34	12	CDS
hsa-miR-6881-5p	NM_033668	ITGB1	.(((((.(((((	946	967	0.923077	-18.7	1	0.080423	0.662	0.264319	1.079791	-5.33701	18	21	11	CDS
hsa-miR-6881-5p	NM_133376	ITGB1	..((((((.((((	631	665	0.846154	-19.2	1	0.001266	0.603	1.703138	0.946588	-6.01339	19	34	12	CDS
hsa-miR-6881-5p	NM_002211	ITGB1	..((((((.((((	514	548	0.846154	-19.2	1	0.001266	0.603	1.390145	1.124102	-6.01339	19	34	12	CDS

miRWalk_miRNA_Targets (8)

Sequence alignment between HSPD1 & hsa-miR-6881-5p

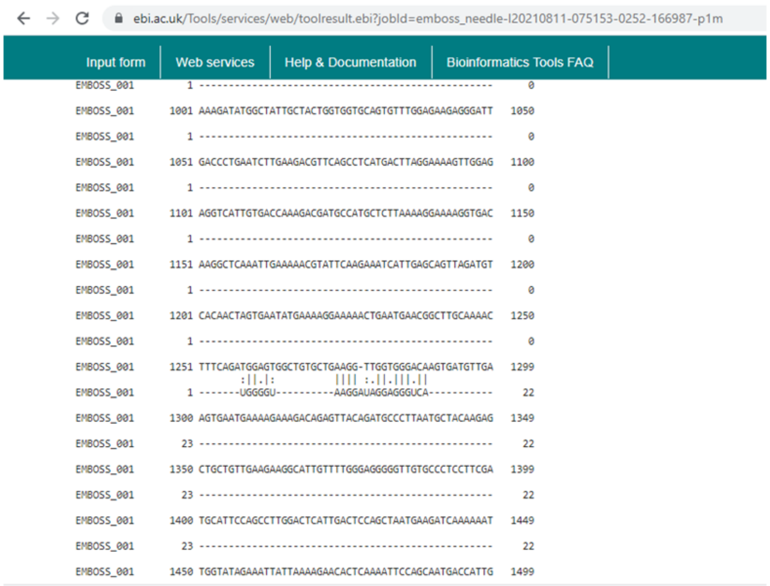

ebi.ac.uk/Tools/services/web/toolresult.ebi?jobId=emboss_needle-I20210811-075153-0252-166987-p1m

Input form | Web services | Help & Documentation | Bioinformatics Tools FAQ

```
EMBOSS_001         1 --------------------------------------------------      0

EMBOSS_001      1001 AAAGATATGGCTATTGCTACTGGTGGTGCAGTGTTTGGAGAAGAGGGATT   1050

EMBOSS_001         1 --------------------------------------------------      0

EMBOSS_001      1051 GACCCTGAATCTTGAAGACGTTCAGCCTCATGACTTAGGAAAAGTTGGAG   1100

EMBOSS_001         1 --------------------------------------------------      0

EMBOSS_001      1101 AGGTCATTGTGACCAAAGACGATGCCATGCTCTTAAAAGGAAAAGGTGAC   1150

EMBOSS_001         1 --------------------------------------------------      0

EMBOSS_001      1151 AAGGCTCAAATTGAAAAACGTATTCAAGAAATCATTGAGCAGTTAGATGT   1200

EMBOSS_001         1 --------------------------------------------------      0

EMBOSS_001      1201 CACAACTAGTGAATATGAAAAGGAAAAACTGAATGAACGGCTTGCAAAAC   1250

EMBOSS_001         1 --------------------------------------------------      0

EMBOSS_001      1251 TTTCAGATGGAGTGGCTGTGCTGAAGG-TTGGTGGGACAAGTGATGTTGA   1299
                            :||.|:          |||| :.||.|||.||
EMBOSS_001         1 -------UGGGGU----------AAGGAUAGGAGGGUCA-----------     22

EMBOSS_001      1300 AGTGAATGAAAAGAAAGACAGAGTTACAGATGCCCTTAATGCTACAAGAG   1349

EMBOSS_001        23 --------------------------------------------------     22

EMBOSS_001      1350 CTGCTGTTGAAGAAGGCATTGTTTTGGGAGGGGGTTGTGCCCTCCTTCGA   1399

EMBOSS_001        23 --------------------------------------------------     22

EMBOSS_001      1400 TGCATTCCAGCCTTGGACTCATTGACTCCAGCTAATGAAGATCAAAAAAT   1449

EMBOSS_001        23 --------------------------------------------------     22

EMBOSS_001      1450 TGGTATAGAAATTATTAAAAGAACACTCAAAATTCCAGCAATGACCATTG   1499
```

Sequence alignment between MMP14 & hsa-miR-6881-5p

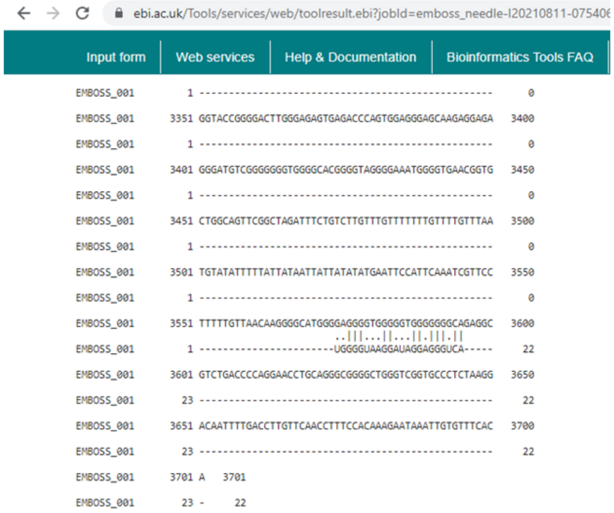

ebi.ac.uk/Tools/services/web/toolresult.ebi?jobId=emboss_needle-I20210811-07540[illegible]

Input form | Web services | Help & Documentation | Bioinformatics Tools FAQ

```
EMBOSS_001         1 --------------------------------------------------      0
EMBOSS_001      3351 GGTACCGGGGACTTGGGAGAGTGAGACCCAGTGGAGGGAGCAAGAGGAGA   3400
EMBOSS_001         1 --------------------------------------------------      0
EMBOSS_001      3401 GGGATGTCGGGGGGGTGGGGCACGGGGTAGGGGAAATGGGGTGAACGGTG   3450
EMBOSS_001         1 --------------------------------------------------      0
EMBOSS_001      3451 CTGGCAGTTCGGCTAGATTTCTGTCTTGTTTGTTTTTTTGTTTTGTTTAA   3500
EMBOSS_001         1 --------------------------------------------------      0
EMBOSS_001      3501 TGTATATTTTTATTATAATTATTATATATGAATTCCATTCAAATCGTTCC   3550
EMBOSS_001         1 --------------------------------------------------      0
EMBOSS_001      3551 TTTTTGTTAACAAGGGGCATGGGGAGGGGTGGGGGTGGGGGGGCAGAGGC   3600
                                            ..|||...||...||.|||.||
EMBOSS_001         1 -----------------------UGGGGUAAGGAUAGGAGGGUCA-----     22
EMBOSS_001      3601 GTCTGACCCCAGGAACCTGCAGGGCGGGGCTGGGTCGGTGCCCTCTAAGG   3650
EMBOSS_001        23 --------------------------------------------------     22
EMBOSS_001      3651 ACAATTTTGACCTTGTTCAACCTTTCCACAAAGAATAAATTGTGTTTCAC   3700
EMBOSS_001        23 --------------------------------------------------     22
EMBOSS_001      3701 A   3701
EMBOSS_001        23 -     22
```

Sequence alignment between ITGB1 & hsa-miR-6881-5p

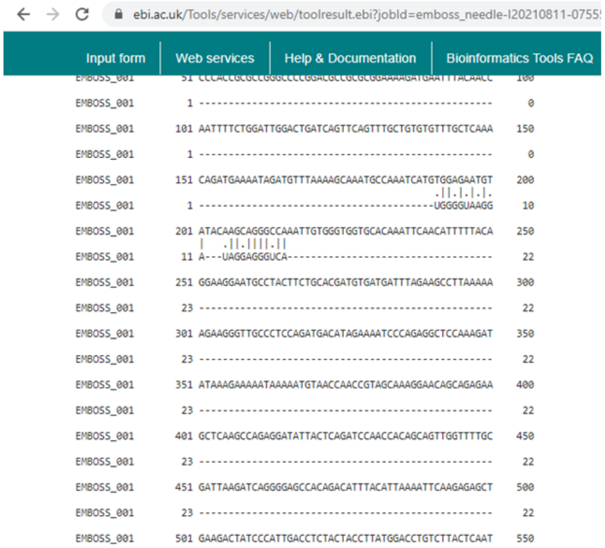

ebi.ac.uk/Tools/services/web/toolresult.ebi?jobId=emboss_needle-I20210811-0755[illegible]

Input form | Web services | Help & Documentation | Bioinformatics Tools FAQ

```
EMBOSS_001        51 [illegible]    100
EMBOSS_001         1 --------------------------------------------------      0
EMBOSS_001       101 AATTTTCTGGATTGGACTGATCAGTTCAGTTTGCTGTGTGTTTGCTCAAA    150
EMBOSS_001         1 --------------------------------------------------      0
EMBOSS_001       151 CAGATGAAAATAGATGTTTAAAAGCAAATGCCAAATCATGTGGAGAATGT    200
                                                             .||.|.|.|.
EMBOSS_001         1 ----------------------------------------UGGGGUAAGG     10
EMBOSS_001       201 ATACAAGCAGGGCCAAATTGTGGGTGGTGCACAAATTCAACATTTTTACA    250
                     |   .||.||||.||
EMBOSS_001        11 A---UAGGAGGGUCA-----------------------------------     22
EMBOSS_001       251 GGAAGGAATGCCTACTTCTGCACGATGTGATGATTTAGAAGCCTTAAAAA    300
EMBOSS_001        23 --------------------------------------------------     22
EMBOSS_001       301 AGAAGGGTTGCCCTCCAGATGACATAGAAAATCCCAGAGGCTCCAAAGAT    350
EMBOSS_001        23 --------------------------------------------------     22
EMBOSS_001       351 ATAAAGAAAAATAAAAATGTAACCAACCGTAGCAAAGGAACAGCAGAGAA    400
EMBOSS_001        23 --------------------------------------------------     22
EMBOSS_001       401 GCTCAAGCCAGAGGATATTACTCAGATCCAACCACAGCAGTTGGTTTTGC    450
EMBOSS_001        23 --------------------------------------------------     22
EMBOSS_001       451 GATTAAGATCAGGGGAGCCACAGACATTTACATTAAAATTCAAGAGAGCT    500
EMBOSS_001        23 --------------------------------------------------     22
EMBOSS_001       501 GAAGACTATCCCATTGACCTCTACTACCTTATGGACCTGTCTTACTCAAT    550
```

Figure S5: Validation of the interaction between the retrieved hsa-miR-6881-5p and lnc-SPARCL1-1:2 lncRNA

Predicted interaction between hsa-miR-6881-5p & lncRNA SPARCL

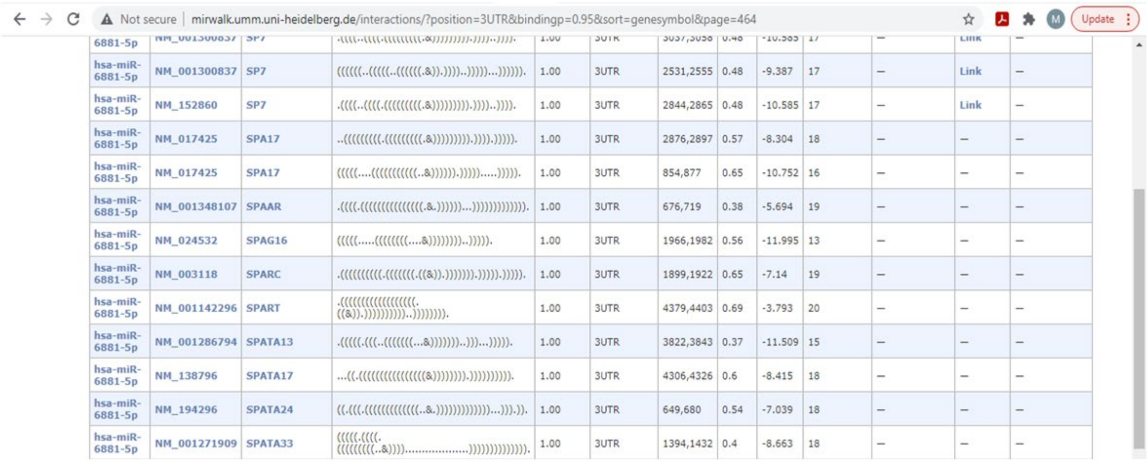

6881-5p	NM_001300837	SP7	[illegible]	1.00	3UTR	3037,3058	0.48	-10.585	17	–	Link	–
hsa-miR-6881-5p	NM_001300837	SP7	((((((..(((((..((((((.&)).)))).)).)))))...)))))).	1.00	3UTR	2531,2555	0.48	-9.387	17	–	Link	–
hsa-miR-6881-5p	NM_152860	SP7	.((((..((((.(((((((((.&))))))))).)))).)))).	1.00	3UTR	2844,2865	0.48	-10.585	17	–	Link	–
hsa-miR-6881-5p	NM_017425	SPA17	..(((((((((.(((((((((.&))))))))).)))).))))).	1.00	3UTR	2876,2897	0.57	-8.304	18	–	–	–
hsa-miR-6881-5p	NM_017425	SPA17	(((((....(((((((((((..&)))))).)))))).....))))).	1.00	3UTR	854,877	0.65	-10.752	16	–	–	–
hsa-miR-6881-5p	NM_001348107	SPAAR	.((((.((((((((((((((((.&.))))))...)))))))))))))).	1.00	3UTR	676,719	0.38	-5.694	19	–	–	–
hsa-miR-6881-5p	NM_024532	SPAG16	(((((.....((((((((....&))))))))..))))).	1.00	3UTR	1966,1982	0.56	-11.995	13	–	–	–
hsa-miR-6881-5p	NM_003118	SPARC	.((((((((((.(((((((.((&)).))))))).))))).))))).	1.00	3UTR	1899,1922	0.65	-7.14	19	–	–	–
hsa-miR-6881-5p	NM_001142296	SPART	.((((((((((((((((((. ((&)).)))))))))))..)))))))).	1.00	3UTR	4379,4403	0.69	-3.793	20	–	–	–
hsa-miR-6881-5p	NM_001286794	SPATA13	.(((((.(((..(((((((...&)))))))..)))...))))).	1.00	3UTR	3822,3843	0.37	-11.509	15	–	–	–
hsa-miR-6881-5p	NM_138796	SPATA17	...((.(((((((((((((((((&))))))))).)))))))))).	1.00	3UTR	4306,4326	0.6	-8.415	18	–	–	–
hsa-miR-6881-5p	NM_194296	SPATA24	((.(((.((((((((((((((..&.))))))))))))))...))).)).	1.00	3UTR	649,680	0.54	-7.039	18	–	–	–
hsa-miR-6881-5p	NM_001271909	SPATA33	(((((.((((. (((((((((..&))))...................))))))))))))).	1.00	3UTR	1394,1432	0.4	-8.663	18	–	–	–

lnc-SPARCL1-1:2 basic information

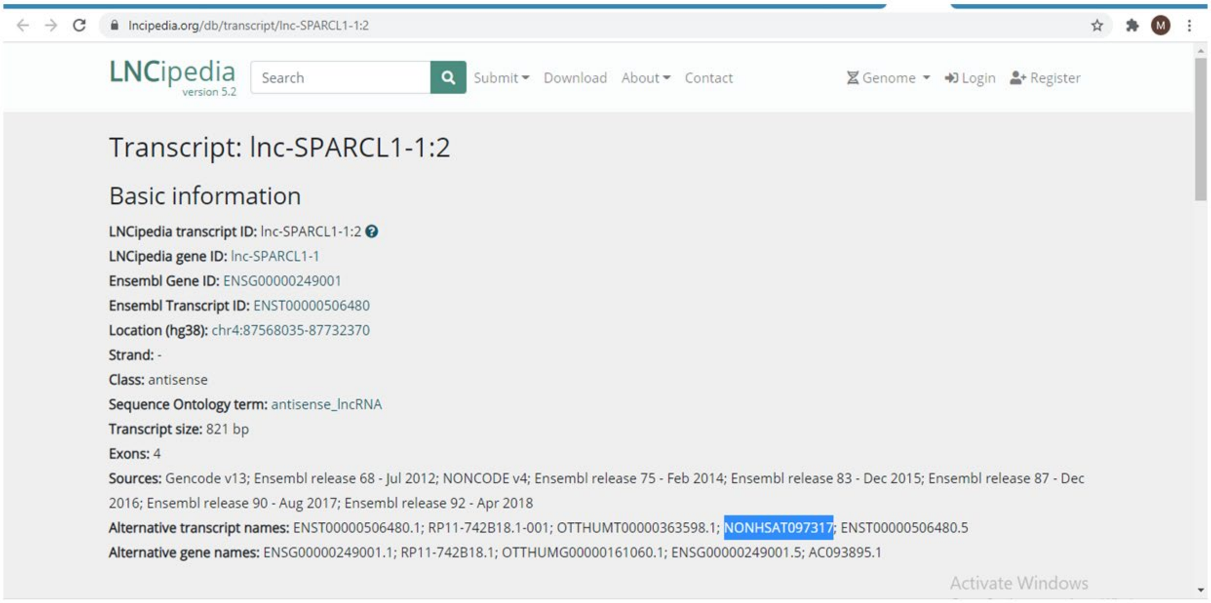

Transcript: lnc-SPARCL1-1:2

Basic information

LNCipedia transcript ID: lnc-SPARCL1-1:2

LNCipedia gene ID: lnc-SPARCL1-1

Ensembl Gene ID: ENSG00000249001

Ensembl Transcript ID: ENST00000506480

Location (hg38): chr4:87568035-87732370

Strand: -

Class: antisense

Sequence Ontology term: antisense_lncRNA

Transcript size: 821 bp

Exons: 4

Sources: Gencode v13; Ensembl release 68 - Jul 2012; NONCODE v4; Ensembl release 75 - Feb 2014; Ensembl release 83 - Dec 2015; Ensembl release 87 - Dec 2016; Ensembl release 90 - Aug 2017; Ensembl release 92 - Apr 2018

Alternative transcript names: ENST00000506480.1; RP11-742B18.1-001; OTTHUMT00000363598.1; NONHSAT097317; ENST00000506480.5

Alternative gene names: ENSG00000249001.1; RP11-742B18.1; OTTHUMG00000161060.1; ENSG00000249001.5; AC093895.1

Sequence alignment between lnc-SPARCL1-1:2 & hsa-miR-6881-5p by European bioinformatics institute database

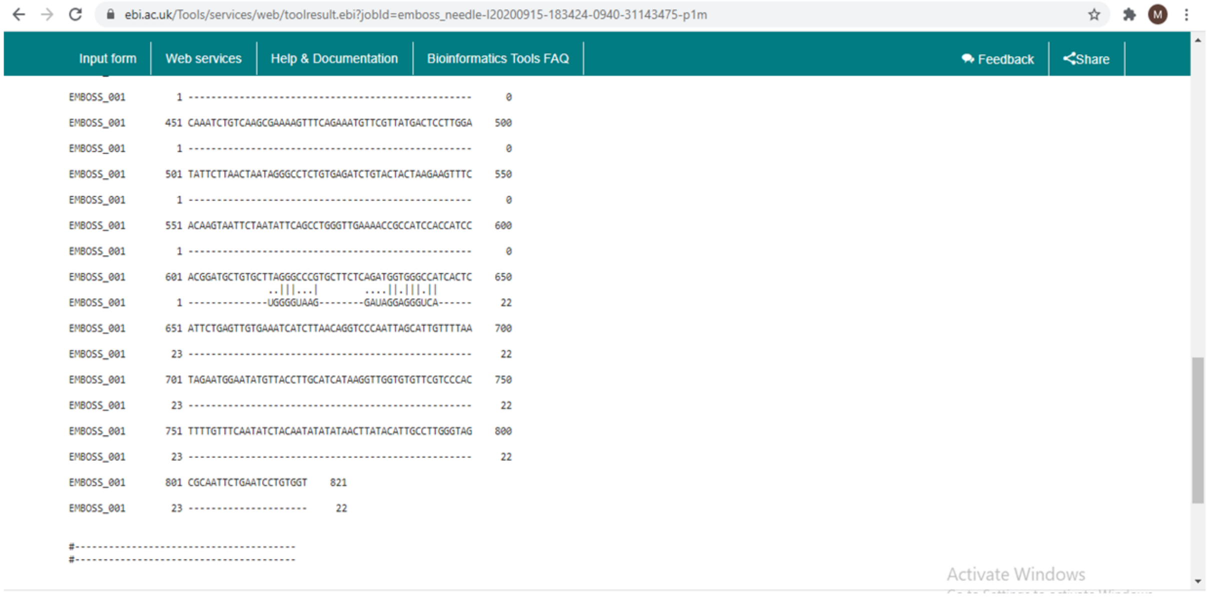

ebi.ac.uk/Tools/services/web/toolresult.ebi?jobId=emboss_needle-I20200915-183424-0940-31143475-p1m

Input form | Web services | Help & Documentation | Bioinformatics Tools FAQ | Feedback | Share

```
EMBOSS_001         1 --------------------------------------------------      0
EMBOSS_001       451 CAAATCTGTCAAGCGAAAAGTTTCAGAAATGTTCGTTATGACTCCTTGGA    500
EMBOSS_001         1 --------------------------------------------------      0
EMBOSS_001       501 TATTCTTAACTAATAGGGCCTCTGTGAGATCTGTACTACTAAGAAGTTTC    550
EMBOSS_001         1 --------------------------------------------------      0
EMBOSS_001       551 ACAAGTAATTCTAATATTCAGCCTGGGTTGAAAACCGCCATCCACCATCC    600
EMBOSS_001         1 --------------------------------------------------      0
EMBOSS_001       601 ACGGATGCTGTGCTTAGGGCCCGTGCTTCTCAGATGGTGGGCCATCACTC    650
                                  ..|||...|        ....||.|||.||
EMBOSS_001         1 -------------UGGGGUAAG--------GAUAGGAGGGUCA------     22
EMBOSS_001       651 ATTCTGAGTTGTGAAATCATCTTAACAGGTCCCAATTAGCATTGTTTTAA    700
EMBOSS_001        23 --------------------------------------------------     22
EMBOSS_001       701 TAGAATGGAATATGTTACCTTGCATCATAAGGTTGGTGTGTTCGTCCCAC    750
EMBOSS_001        23 --------------------------------------------------     22
EMBOSS_001       751 TTTTGTTTCAATATCTACAATATATATAACTTATACATTGCCTTGGGTAG    800
EMBOSS_001        23 --------------------------------------------------     22
EMBOSS_001       801 CGCAATTCTGAATCCTGTGGT    821
EMBOSS_001        23 ---------------------     22


#---------------------------------------
#---------------------------------------
```

Sequence alignment between lnc-SPARCL1-1:2 & hsa-miR-6881-5p by lnctar tool

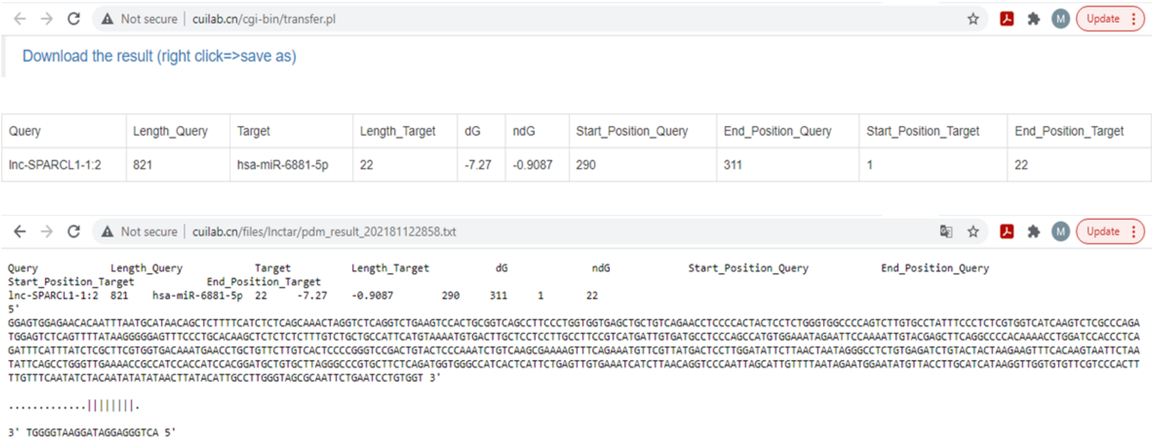

Not secure | cuilab.cn/cgi-bin/transfer.pl

Download the result (right click=>save as)

Query	Length_Query	Target	Length_Target	dG	ndG	Start_Position_Query	End_Position_Query	Start_Position_Target	End_Position_Target
lnc-SPARCL1-1:2	821	hsa-miR-6881-5p	22	-7.27	-0.9087	290	311	1	22

Not secure | cuilab.cn/files/lnctar/pdm_result_202181122858.txt

```
Query            Length_Query          Target          Length_Target          dG            ndG            Start_Position_Query            End_Position_Query
Start_Position_Target            End_Position_Target
lnc-SPARCL1-1:2  821    hsa-miR-6881-5p  22     -7.27    -0.9087        290     311     1       22
5'
GGAGTGGAGAACACAATTTAATGCATAACAGCTCTTTTCATCTCTCAGCAAACTAGGTCTCAGGTCTGAAGTCCACTGCGGTCAGCCTTCCCTGGTGGTGAGCTGCTGTCAGAACCTCCCCACTACTCCTCTGGGTGGCCCCAGTCTTGTGCCTATTTCCCTCTCGTGGTCATCAAGTCTCGCCCAGA
TGGAGTCTCAGTTTTATAAGGGGGAGTTTCCCTGCACAAGCTCTCTCTCTTTGTCTGCTGCCATTCATGTAAAATGTGACTTGCTCCTCCTTGCCTTCCGTCATGATTGTGATGCCTCCCAGCCATGTGGAAATAGAATTCCAAAATTGTACGAGCTTCAGGCCCCACAAAACCTGGATCCACCCTCA
GATTTCATTTATCTCGCTTCGTGGTGACAAATGAACCTGCTGTTCTTGTCACTCCCCGGGTCCGACTGTACTCCCAAATCTGTCAAGCGAAAAGTTTCAGAAATGTTCGTTATGACTCCTTGGATATTCTTAACTAATAGGGCCTCTGTGAGATCTGTACTACTAAGAAGTTTCACAAGTAATTCTAA
TATTCAGCCTGGGTTGAAAACCGCCATCCACCATCCACGGATGCTGTGCTTAGGGCCCGTGCTTCTCAGATGGTGGGCCATCACTCATTCTGAGTTGTGAAATCATCTTAACAGGTCCCAATTAGCATTGTTTTAATAGAATGGAATATGTTACCTTGCATCATAAGGTTGGTGTGTTCGTCCCACTT
TTGTTTCAATATCTACAATATATATAACTTATACATTGCCTTGGGTAGCGCAATTCTGAATCCTGTGGT 3'

.............||||||||.

3' TGGGGTAAGGATAGGAGGGTCA 5'
```

Interaction between lnc-SPARCL1-1:2 & HSPD1, MMP14, ITGB1 by enrichr tool

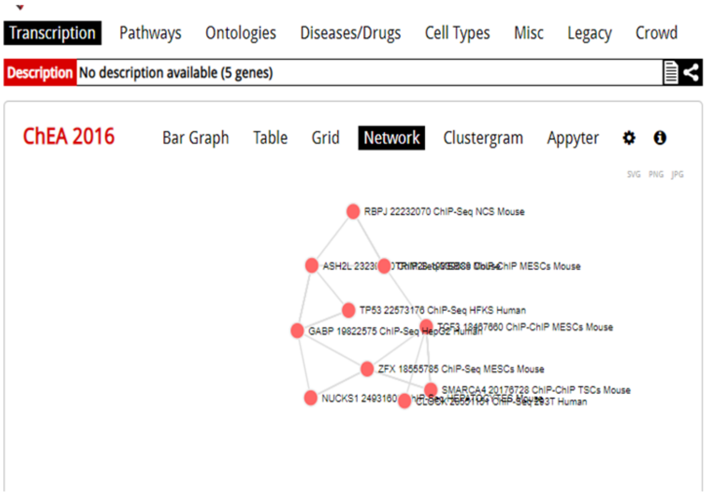

Supplement: Supplementary file 1 [file biomedicines-09-01248-s001.zip › biomedicines-1385152-supplementary.pdf]
